# The *P. aeruginosa* effector Tse5 forms membrane pores disrupting the membrane potential of intoxicated bacteria

**DOI:** 10.1038/s42003-022-04140-y

**Published:** 2022-11-05

**Authors:** Amaia González-Magaña, Jon Altuna, María Queralt-Martín, Eneko Largo, Carmen Velázquez, Itxaso Montánchez, Patricia Bernal, Antonio Alcaraz, David Albesa-Jové

**Affiliations:** 1 Fundación Biofísica Bizkaia/Biofisika Bizkaia Fundazioa (FBB) and Departamento de Bioquímica y Biología Molecular, Instituto Biofisika (CSIC, UPV/EHU), University of the Basque Country, 48940 Leioa, Spain; 2 Laboratory of Molecular Biophysics, Department of Physics, University Jaume I, 12071 Castellón, Spain; 3 Departamento de Inmunología, Microbiología y Parasitología, University of the Basque Country, 48940 Leioa, Spain; 4 Departamento de Microbiología, Facultad de Biología, Universidad de Sevilla, 41012 Sevilla, Spain; 5 Ikerbasque, Basque Foundation for Science, 48013 Bilbao, Spain

**Keywords:** Pathogens, Permeation and transport, Bacterial toxins

## Abstract

The type VI secretion system (T6SS) of *Pseudomonas aeruginosa* injects effector proteins into neighbouring competitors and host cells, providing a fitness advantage that allows this opportunistic nosocomial pathogen to persist and prevail during the onset of infections. However, despite the high clinical relevance of *P. aeruginosa*, the identity and mode of action of most *P. aeruginosa* T6SS-dependent effectors remain to be discovered. Here, we report the molecular mechanism of Tse5-CT, the toxic auto-proteolytic product of the *P. aeruginosa* T6SS exported effector Tse5. Our results demonstrate that Tse5-CT is a pore-forming toxin that can transport ions across the membrane, causing membrane depolarisation and bacterial death. The membrane potential regulates a wide range of essential cellular functions; therefore, membrane depolarisation is an efficient strategy to compete with other microorganisms in polymicrobial environments.

## Abstract (web summary)

The *Pseudomonas aeruginosa* Type 6 secretion effector Tse5 forms pores in the cytoplasmic membrane when ectopically produced and hence has a bacteriolytic effect by depolarising the inner membrane potential.

## Introduction

Bacteria usually associate with forming polymicrobial structures called biofilms^1,2^, where they frequently compete with other microorganisms for space and nutrients. In this context, many Gram-negative bacteria employ the type VI secretion system (T6SS) to deliver toxic effectors to close competitors to either kill them or subvert their key biological functions. Thus, the T6SS provides an evolutionary advantage to bacteria that allows them to thrive and succeed in niche colonisation^3–5^. The T6SS assembles inside bacteria from 14 essential components related to the tail proteins of bacteriophage T4^5–11^. The injection mechanism is energised by the contraction of the TssBC sheath (also known as VipAB)^10,12,13^, which encapsulates the Hcp tubular structure. The Hcp tube is topped by the VgrG–PAAR tip complex, which is believed to facilitate the puncture of the producer and target cell membranes upon TssBC sheath contraction^9,10^. T6SS effectors are either encapsulated within the Hcp tube^14^ or associated with the VgrG–PAAR tip complex^15^. In addition, other effectors exist as extension domains on VgrG^16–18^, Hcp^19^, or PAAR proteins^10^. Importantly, bacteria that have a specific antibacterial T6SS effector also encode a corresponding cognate immunity protein, which specifically binds to the effector, thereby neutralising the effector’s toxicity^20^.

*P. aeruginosa* contains in its genome three independent T6SS clusters (H1, H2, and H3-T6SS)^21,22^. Known effectors delivered by the H1-T6SS have an antiprokaryotic activity that target: (i) the cell wall peptidoglycan (Tse1: peptidase activity^23,24^ and Tse3: muramidase activity^25,26^), (ii) the membrane (Tse4^14,27^), (iii) NAD(P)+ (Tse6: glycohydrolase activity^28^), (iv) the DNA (Tse7: DNase activity^29^), and (v) protein biosynthesis (Tse8^30^). Furthermore, a key to deciphering the role of T6SS effectors is to understand its regulation mechanisms. Bacteria have evolved different strategies for deploying their T6SS. Whereas some strains of *Vibrio cholerae* assemble and fire their apparatus at random locations in the cell, *P. aeruginosa* PAO1 uses the H1-T6SS more defensively, assembling and firing it only exactly where it detects an attack by another bacterium^31–37^. Consequently, while *V. cholerae* is outcompeted in cocultures with *P. aeruginosa* when both organisms have a functioning T6SS, *P. aeruginosa* does not kill a T6SS-negative *V. cholerae* strain^36^. This defensive mechanism has been observed in cocultures of *P. aeruginosa* with other T6SS+ bacterial species, including *Acinetobacter baylyi* or *Burkholderia thailandensis*^36,38,39^ and was termed the T6SS tit-for-tat response^36^.

In the present study, we investigate Tse5 (PA2684), an H1-T6SS-dependent effector conserved among *P. aeruginosa* strains isolated from patients with cystic fibrosis and bronchiectasis^40^. Two laboratories^14,41^, almost simultaneously, discovered Tse5. Nonetheless, its mechanism of action remains elusive. It was demonstrated that Tse5-CT is toxic when expressed in the cytoplasm of *Escherichia coli*^41^ and when directed to its periplasm^14^. Tse5 associates with VgrG4 (PA2685, also known as VgrG1c) for H1-T6SS-dependent delivery into target cells^14,41^. Furthermore, Tse5-producing cells protect themselves from intoxication by the cognate immunity protein Tsi5 (PA2683)^14,41^. Tsi5 contains two predicted transmembrane regions. Furthermore, subcellular localisation experiments and Western blot analysis have shown that it fractionates with the cytoplasmic membrane of *E. coli* cells expressing Tsi5, which supports the hypothesis that Tsi5 is an integral membrane protein^41^.

Bioinformatic analysis of Tse5 predicts three domains: An N-terminus domain (residues Met1-Lys47) that is predicted to fold like a PAAR domain that lacks the signature Pro–Ala–Ala–Arg motif, a large central domain (Pro48-Leu1168) with homology to Rearrangement hot spot repeats (PF05593) (Rhs repeats)^42^, and a C-terminal region (Tse5-CT; residues Ile1169–Gln1317) with no predicted function. Notably, the PAAR motif is conserved in many putative toxins, including Rhs toxins, implicated in T6SS-dependent interbacterial competition^43–48^. Rhs toxins are bacterial exotoxins that belong to the polymorphic category^49^. They are large proteins of usually more than 1500 residues with variable C-terminal toxic domains, and they are ubiquitous in Gram-negative and Gram-positive bacteria^50^. Flanking the N-terminus of the C-terminal toxic domain, there is a conserved DPXGL-(18)-DPXGL motif, which marks the limit of the Rhs core domain. This conserved motif forms the active site of an aspartyl protease that releases the C-terminal toxic domain^51,52^.

The C-terminus region of Tse5 (Tse5-CT; residues Ile1169–Gln1317) harbours the Tse5 toxicity^14,41^. However, Tse5-CT has no predicted function, which challenges the elucidation of its mechanism of action.

## Results

### Tse5-CT expression has a bacteriolytic effect on *Pseudomonas putida* cells that can be reversed by co-expression of the cognate immunity protein Tsi5

**Fig. 1:**
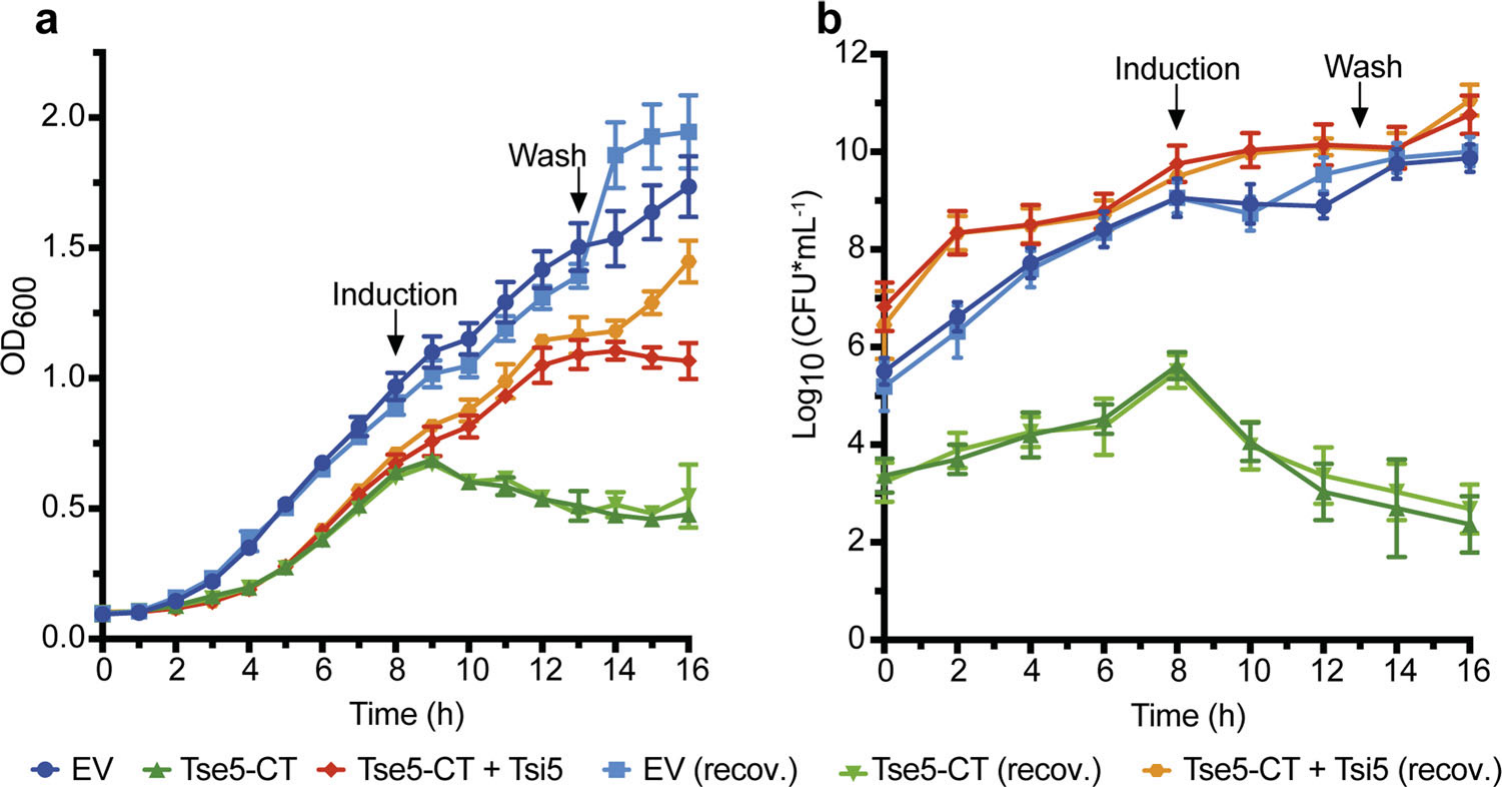
Tse5-CT has a bacteriolytic effect when expressed in *P. putida*. **a** Monitoring growth (OD_600_) in LB medium of *P. putida* cells harbouring pS238D1 empty vector (EV) as a negative control or plasmids directing the expression of Tse5-CT, or Tse5-CT and Tsi5 together. Expression of Tse5-CT and Tsi5 was induced after 8 h of bacterial growth (see the arrow) with 1 mM m-Toluic acid (TA) and 0.1 mM isopropyl 1-thio-β-d-galactopyranoside (IPTG), respectively. Co-expression of Tse5-CT and Tsi5 was induced by adding to the media both TA and IPTG. To stop the induction, the recovered cells (recov.) were washed and resuspended in fresh LB without inductors (see the arrow). Expression of Tse5-CT caused inhibition of bacterial growth, and cells did not recover the capacity to duplicate after washing. Tsi5 confers cell protection from Tse5 expression. **b** Monitoring growth on solid medium. Samples of *P. putida* growing cells were taken every 2 h, and dilutions were spotted on LB agar plates. Colony-forming units (CFU) were counted 24 h later. All measurements were made in triplicate (*n* = 3 biological replicates). The graphs show the mean values and ± standard deviations (SD). Some error bars are not visible due to overlaps with symbols.

To provide insight into the mode of action of Tse5-CT, we first evaluated if it can kill *P. putida* EM383 cells (bacteriolytic effect) or rather cause a transient growth inhibition (bacteriostatic effect). The *P. putida* strain EM383 results from 11 deletions introduced in the genome of *P. putida* strain KT2440 to optimise heterologous gene expression^53^. We observed that inducing the expression of Tse5-CT resulted in a bacterial growth arrest, which, 8 h after Tse5-CT induction, resulted in an OD_600_ drop of 0.16(3) arb. unit (Fig. 1a), and a CFU drop of 3.2(9) log CFU mL^−1^ (Fig. 1b). Furthermore, we failed to recover bacterial growth when the inducer (m-toluic acid) was removed 5 h post-induction. On the contrary, bacteria transformed with the empty vector (pS238D1) grew as expected, reaching OD_600_ values of 1.7(1) and CFUs of 9.9(3) CFU mL^−1^. This result would indicate that Tse5-CT has a bacteriolytic effect when expressed in *P. putida* cells.

Co-expression of Tsi5 (red/orange curves) could reverse the Tse5-CT phenotype (green curves). Following co-induction of Tse5-CT and Tsi5 with m-toluic acid and isopropyl-β-d-1-thiogalactopyranoside (IPTG), respectively, bacterial cultures continue to grow at a normal rate (Fig. 1a) to reach CFU values comparable to control bacteria (blue curves; Fig. 1b). Previous studies were also able to show Tsi5 (RhsI1) protection when Tse5 and Tsi5 were co-expressed in *E. coli*^54^. In order to be able to reverse the Tse5-CT phenotype in *P. putida*, we had to reduce the IPTG concentration from 1 to 0.1 mM, which might be associated with difficulties in producing transmembrane proteins in a heterologous host^14,55^.

### Tse5-CT toxicity causes depolarisation of intoxicated *Pseudomonas putida* cells that can be reversed by co-expression of the cognate immunity protein Tsi5

Tsi5 contains two predicted transmembrane regions. Furthermore, subcellular localisation experiments and Western blot analysis have shown that Tsi5 fractionates with the cytoplasmic membrane of *E. coli* cells expressing Tsi5^41^, which altogether supports the hypothesis that Tsi5 is an integral membrane protein that inserts in the cytoplasmic membrane of *P. aeruginosa*. Moreover, given that bioinformatic analysis of Tse5-CT did not predict any enzymatic activity, we hypothesised that Tse5-CT might target the bacterial cytoplasmic membrane, which might affect the cell permeability or the membrane potential of intoxicated cells.

The possible Tse5-CT effect on cellular permeability or membrane potential was evaluated by flow cytometry using *P. putida* EM383 cells. To define cell populations, we performed various controls (see the “Methods” section for details; Supplementary Figs. 2, 3). A depolarisation positive control was obtained by treating cells with the antibiotic polymyxin B (Supplementary Fig. 2b). For a permeabilisation positive control, we treated cells with a heat shock (Supplementary Fig. 2c). A double positive control was performed by treating cells with heat shock and polymyxin B (Supplementary Fig. 2d). Finally, negative controls consist of *P. putida* cells transformed with the empty plasmids (Supplementary Fig. 3).

*P. putida* cells were transformed with the plasmid coding for Tse5-CT wild type (pS238D1::*tse5-CT*) or a variant encoding for the pelB leader sequence (pS238D1::*sptse5-CT*), which should direct Tse5-CT to the Sec pathway for translocation into the periplasmic space^56^. Cells were cultured in liquid media, with the expression of Tse5-CT or spTse5-CT induced with m-toluic acid. Ninety minutes after induction, cells were stained with DiBAC_4_(3) and Sytox^TM^ Deep Red. DiBAC_4_(3) is an anionic probe that can permeate into depolarised cells, where it binds to intracellular proteins or the membrane, increasing green fluorescence^57^. Sytox^TM^ Deep Red is a nucleic acid stain that readily penetrates cells with compromised plasma membranes (permeabilised cells), increasing red fluorescence.

**Fig. 2:**
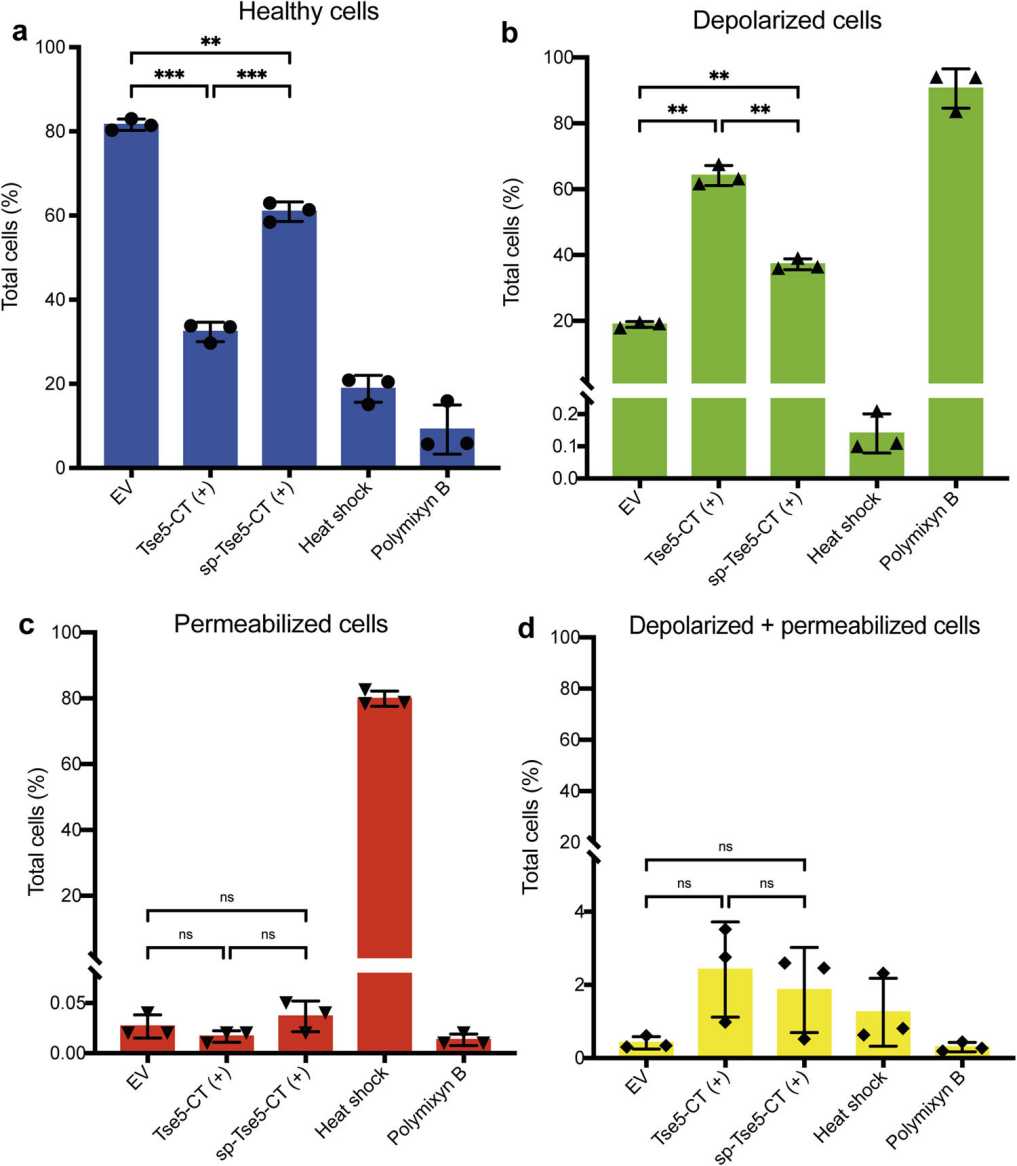
Tse5-CT causes membrane depolarisation when expressed in *P. putida*. Flow cytometry experiments were performed with *P. putida* cells harbouring pS238D1 empty vector (EV) as a negative control and plasmids directing the expression of Tse5-CT or sp-Tse5-CT. Each graph includes two positive controls: cells treated with a heat shock that results in cell permeabilization and cells treated with polymyxin B, which results in cell depolarisation. **a** Flow cytometry results showing healthy cell populations. Healthy cells are not marked by any fluorophore. **b** Flow cytometry data show depolarised cell populations. Depolarised cells are stained with DiBAC_4_(3). **c** Flow cytometry results show permeabilized cell populations. Permeabilized cells are stained with Sytox™ Deep Red. **d** Flow cytometry results show permeabilized and depolarised cell populations. All measurements were made in triplicate (*n* = 3 biological replicates). The graphs show the mean values and ±standard deviations (SD). The one-way ANOVA (Brown–Forsythe ANOVA test) with Dunnett´s T3 multiple comparisons test was used to determine whether there is a significant difference between the mean values of our independent groups (non-significant [ns] if *p* > 0.05, * if *p* ≤ 0.05, ** if *p* ≤ 0.01, *** if *p* ≤ 0.001).

Cells expressing Tse5-CT and treated with DiBAC_4_(3) and Sytox^TM^ Deep Red were analysed by flow cytometry (Fig. 2; Supplementary Fig. 3b). The measurements revealed that Tse5-CT expression resulted in approximately (ca.) 51% reduction of the healthy cell population compared to the healthy cells observed in the negative control (cells transformed with an empty vector; Fig. 2a). However, the decrease in the number of healthy cells is compensated by ca. 46% increase in depolarised cells (Fig. 2b) and ca. 5% increase in cells depolarised and permeabilised (Fig. 2d).

Cells expressing the Tse5-CT variant containing the pelB leader sequence (spTse5-CT) and treated with DiBAC_4_(3) and Sytox^TM^ Deep Red were also analysed by flow cytometry (Fig. 2). In this case, there was ca. 23% reduction in the healthy cell population compared to the healthy cells observed in the negative control (Fig. 2a). Similar to what we observed in cells expressing Tse5-CT, the decrease in healthy cells is mainly compensated by increased depolarised cells (ca. 21% increase in depolarised cells (Fig. 2b), and ca. 2% increase in depolarised and permeabilised cells (Fig. 2d).

These results indicate that Tse5-CT and spTse5-CT change the membrane potential of intoxicated cells but do not considerably affect the integrity of the cytoplasmic membrane. Furthermore, the impact of Tse5-CT seems to be increased in the absence of the signal peptide.

**Fig. 3:**
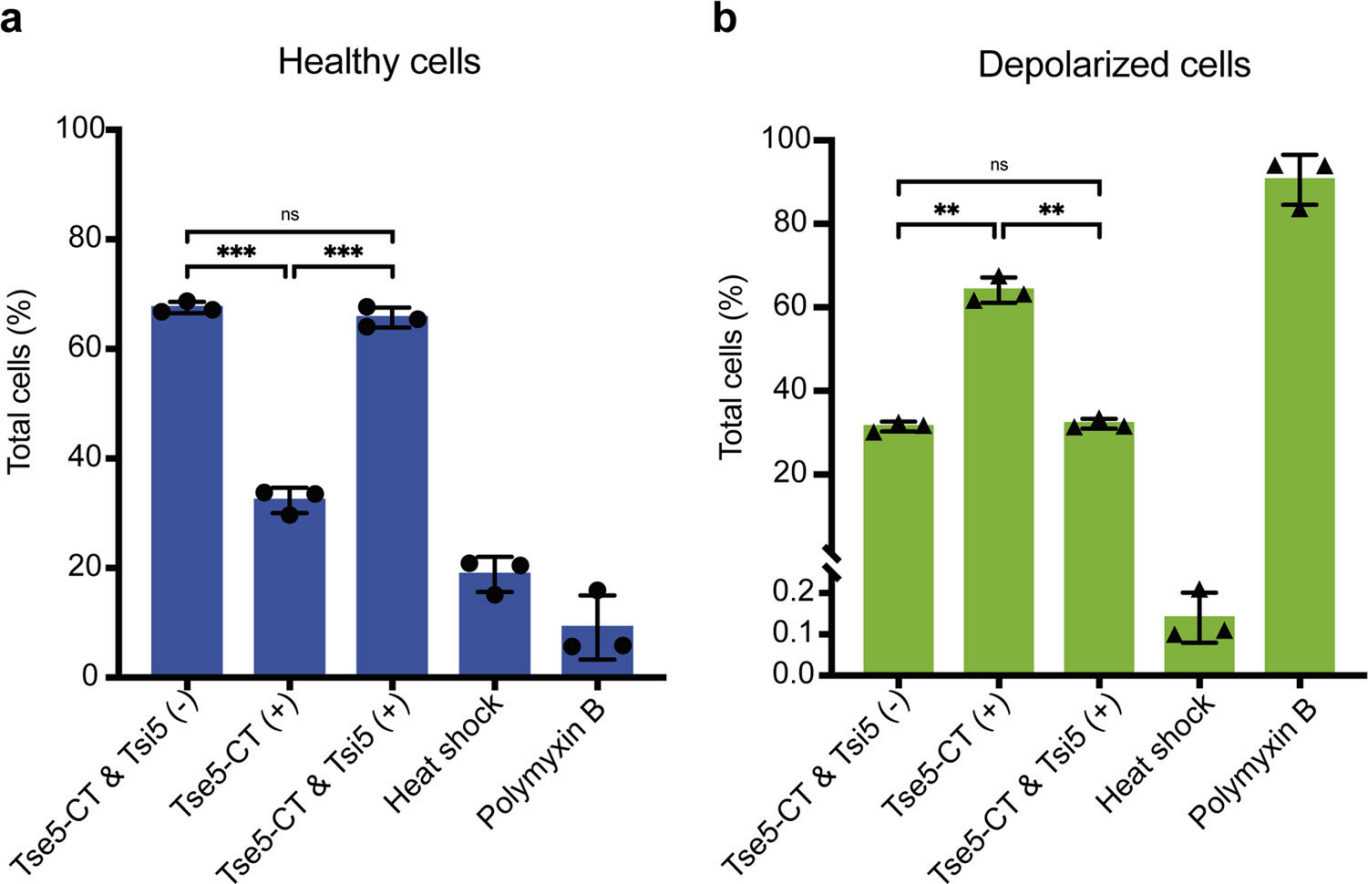
Tsi5 can protect from Tse5-induced membrane depolarisation. Flow cytometry experiments were performed with *P. putida* cells harbouring plasmids directing the expression of Tse5-CT (Tse5-CT^+^) or Tse5-CT and Tsi5 (Tse5-CT^+^ and Tsi5^+^). As a negative control, the graphs include the flow cytometry results of cells transformed with pS238D1::*tse5-CT* and pSEVA424::*tsi5* plasmids and without inducing the expression of the proteins (Tse5-CT^−^ and Tsi5^−^). Each graph includes two positive controls: cells treated with a heat shock that results in cell permeabilization and cells treated with polymyxin B, which results in cell depolarisation. **a** Flow cytometry results showing healthy cell populations. Healthy cells are not marked with any fluorophore. **b** Flow cytometry data show depolarised cell populations. Depolarised cells stain with DiBAC_4_(3). All measurements were made in triplicate (*n* = 3 biological replicates). The graphs show the mean values and ± standard deviations (SD). The one-way ANOVA (Brown–Forsythe ANOVA test) with Dunnett´s T3 multiple comparisons test was used to determine whether there is a significant difference between the mean values of our independent groups (non-significant [ns] if *p* > 0,05, * if *p* ≤ 0.05, ** if *p* ≤ 0.01, *** if *p* ≤ 0.001).

Furthermore, co-expression of Tsi5 can revert the cell depolarisation phenotype induced by Tse5-CT. That is, the cell depolarisation effect is not significantly different between cells co-expressing Tse5-CT and Tsi5 and cells transformed with empty vectors (see the “Methods” section for details; Fig. 3, Supplementary Fig. 3c, d).

### Tse5-CT is a transmembrane protein that spontaneously inserts into model membranes

To evaluate the capacity of Tse5-CT to insert into biological membranes, we first extracted Tse5-CT from purified Tse5 (see experimental details in the “Methods” section; Supplementary Fig. 4). Tse5-CT results from the auto-cleavage of the full-length protein. Flanking the N-terminus of Tse5-CT, there is a conserved DPXGL-(18)-DPXGL motif, which marks the limit of the Rhs core domain and the start of Tse5-CT. This conserved motif, highlighted in Supplementary Fig. 4b, forms the active site of a putative aspartyl protease that could be responsible for cleaving the C-terminal toxic domain^51,52^. In agreement with previous results, two single-point mutants of nucleophilic residues within the putative aspartyl protease domain (D1141A and D1164A) confirmed these two residues are essential for the auto-cleavage of Tse5-CT (Supplementary Fig. 4f).

**Fig. 4:**
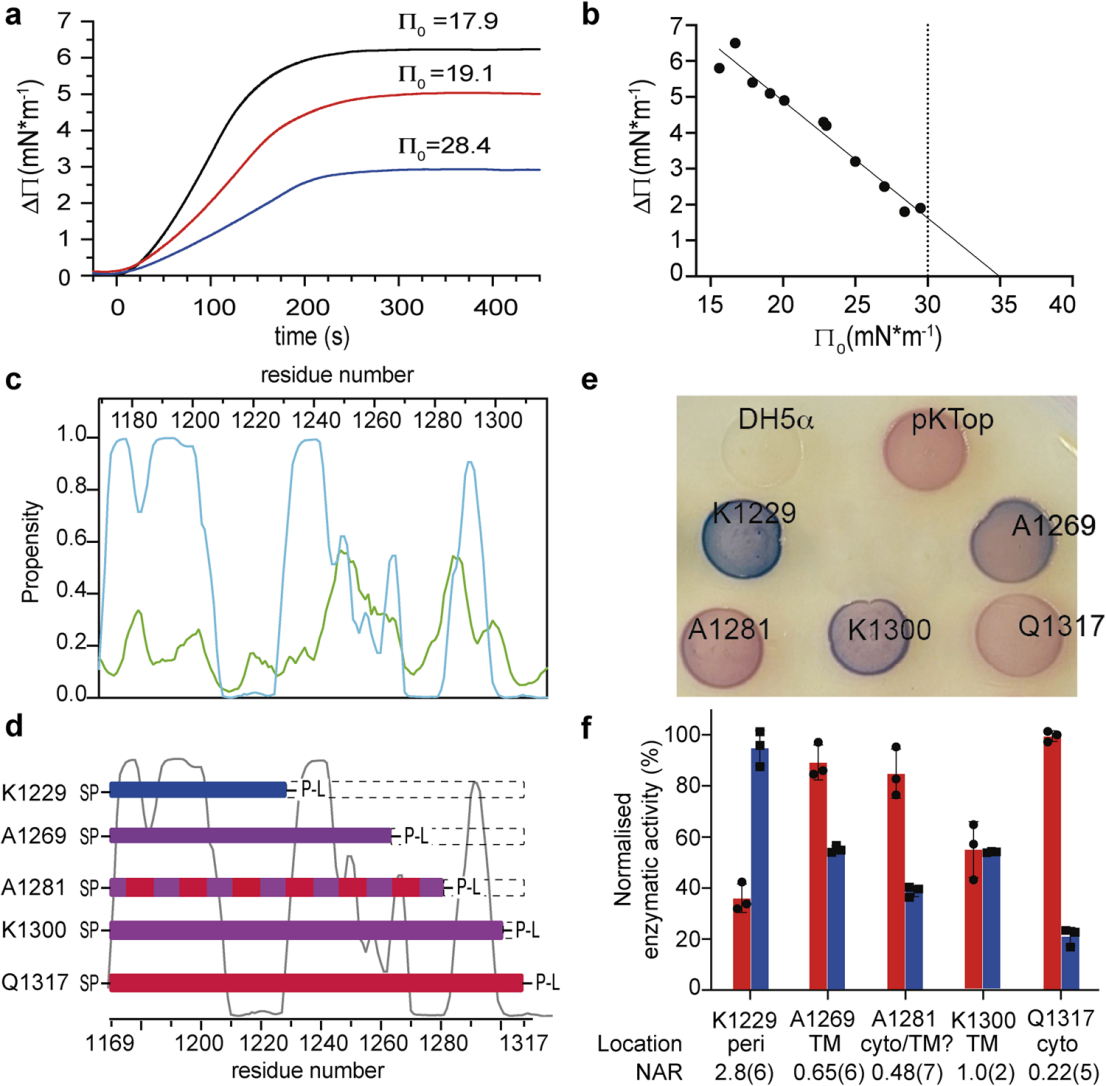
Tse5-CT contains transmembrane regions that allow it to insert into lipid monolayers and the cytoplasmic membrane of *E. coli* cells. **a** Representative Langmuir–Blodgett balance experiment showing the lateral pressure increase on lipid monolayers after the addition of Tse5-CT at time 0. Initial lateral pressures (Π_0_) in mN m^−1^ for representative experiments are indicated above each curve. **b** The plot of lateral pressure increases as a function of initial lateral pressure for every single experiment (*n* = 11). A maximal insertion pressure (MIP) of 34.99 mN m^−1^ has been determined by extrapolating the fitted curve to ΔΠ = 0. The dotted line indicates the threshold value of lateral pressure consistent with unstressed biological membranes. The equation obtained from the linear regression analysis is *y* = −0.3258*x* + 11,401 with an *R*-squared of 0.96. **c** Tse5-CT propensity to contain transmembrane (blue) and amphipathic (green) helices as predicted by MemBrain 3.1. **d** Representation not to scale of constructs containing the dual reporter PhoA-LacZα (abbreviated P-L in the figure) at different C-terminal fusion points of Tse5-CT: K1229, A1269, A1281, K1300 and Q1317 (full-length Tse5-CT). All constructs start at Ile1169, and the truncation points are indicated by the length of the coloured bars and the residue number shown below. The dotted boxes indicate the regions that have been deleted. The predicted transmembrane propensity of Tse5-CT residues is also shown in the background. All fusion constructs contain a signal peptide (SP) at the N-terminal. Bars are colour coded based on the experimental results. Thus, a blue bar corresponds to periplasmic localisation of PhoA-LacZα; a red bar corresponds to a cytoplasmic localisation; a purple bar corresponds to localisation in a TM region; and a red and purple bar (A1281) corresponds to a borderline result, where it is not clear if it localises in the cytosol or a TM region. **e** *E. coli* DH5α cells transformed with spTse5-CT-PhoA-LacZα fusion proteins growing on dual reporter agar plates. **f** Measures of the PhoA-LacZ enzymatic activities for each spTse5-CT-PhoA-LacZα fusion protein. Red and blue bars indicate LacZ and PhoA enzymatic activities, respectively. Calculated NAR values and the location of the dual reporter fusion points are indicated below the graph. All enzymatic activities were measured in triplicate (*n* = 3), and the graph shows the mean values and standard deviations (SD).

Following the purification of Tse5-CT, we measured its ability to partition in lipid monolayers reconstituted from an *E. coli* lipid extract using the Langmuir–Blodgett balance^58^ (Fig. 4a, b). This technique records the insertion of the peptide into the monolayer as an increase in lateral pressure (ΔΠ) from an adjusted initial lateral pressure (Π_0_). Peptide insertion decreases as the initial lateral pressure increases (Fig. 4a) until the critical lateral pressure (Π_c_) is reached, at which the peptide is no longer able to insert into the monolayer. The lipid packing in the outer monolayer of biological membranes approaches lateral surface pressures between 30 and 35 mN/m^59,60^. Thus, a critical lateral pressure in this range upon protein addition indicates that it is able to insert into biological membranes.

Tse5-CT insertion into the lipid monolayer yielded a Π_c_ near to 35 mN/m (Fig. 4b). Therefore, this result indicates that Tse5-CT can spontaneously partition into the hydrophobic core of a lipid monolayer when introduced into a polar buffer, suggesting that it might also be able to insert into biological membranes when delivered by the H1-T6SS into the periplasm/cytoplasm of intoxicated bacteria.

Hydrophobic transmembrane (TM) regions should mediate the insertion of Tse5-CT into biological membranes. Indeed, the prediction of TM helices^61^ suggests that Tse5-CT might contain several putative TM helices (TMH) and amphipathic helices (AH) to insert into biological membranes (Fig. 4c). To evaluate if Tse5-CT includes TM regions, we engineered a series of Tse5-CT deletion mutants fused to a C-terminal dual reporter protein (Fig. 4d). The dual reporter gene is a chimaera containing an *Escherichia coli* alkaline phosphatase (PhoA) gene and the α-peptide gene fragment of the β-galactosidase (LacZα). This PhoA-LacZα dual reporter has been extensively used to study in vivo membrane protein topology by the gene fusion approach^62,63^.

When fused to periplasmic domains of polytopic membrane proteins, the dual reporter gene produces fusion proteins with high PhoA activity and low LacZ activity, and when linked to cytoplasmic domains, it makes fusions with high LacZ activity, but low PhoA activity in *E. coli* strains capable of α-complementation. Thus, the growth of *E. coli* on dual indicator agar plates allows for discrimination between colonies expressing cytoplasmic or periplasmic fusion proteins. On such agar plates, cells with phosphatase activity can convert the X-Pho (5-bromo-4-chloro-3-indolyl-phosphate) substrate into a blue-coloured, precipitated compound, while cells with β-galactosidase activity transform the Red-Gal (6-chloro-3-indolyl-β-d-galactoside) substrate into an insoluble red chromophore. Furthermore, both enzymatic activities can be quantified in permeabilised intact cells using the colourimetric substrates ortho-nitrophenyl-β-d-galactoside (ONPG) and *p*-nitrophenyl phosphate (pNPP) for LacZ and PhoA activities, respectively^64^.

The dual reporter strategy represents a straightforward method for normalising PhoA and LacZ enzymatic activity without determining protein synthesis rates^62^. The initial hypothesis proposed was that the level of expression of a specific membrane protein/PhoA-LacZ fusion would impact the activity levels of both phosphatase and β-galactosidase enzymes, but not their relative ratio^62^. Therefore, given a set of fusion proteins, the phosphatase and β-galactosidase enzymatic activities of each hybrid protein can be normalised to the highest activity values observed within the set of fusions to obtain a normalised activity ratio (NAR). Thus, this ratio provides readily interpretable information about the subcellular location of the particular fusion point (i.e., the residue of the membrane protein after which the PhoA-LacZα is fused). Those values with NAR (${A}_{{{\rm {PhoA}}}}/{{{\rm {Highest}}\; A}}_{{{\rm {PhoA}}}}$:$\,{A}_{{{\rm {LacZ}}}}/{{{\rm {Highest}}\; A}}_{{{\rm {LacZ}}}}$) of greater than 2:1 or less than 1:2 indicate that 67% or more of the reporter activity is properly localised in the periplasm or the cytosol, respectively^62^.

In particular, we engineered five spTse5-CT-PhoA-LacZα fusion proteins (Fig. 4d). Each fusion protein contains different fragments of Tse5-CT. The fusion protein spTse5-CT_1169–1229_-PhoA-LacZα (K1229) contains the first 61 N-terminal residues, spTse5-CT_1169–1269_-PhoA-LacZα (A1269) contains the first 101 residues, spTse5-CT_1169–1281_-PhoA-LacZα (A1281) contains the first 113 residues, spTse5-CT_1169–1300_-PhoA-LacZα (K1300) contains the first 132 residues, and spTse5-CT_1169–1317_-PhoA-LacZα (Q1317) contains the full-length Tse5-CT (149 residues). Furthermore, all the Tse5-CT-PhoA-LacZα fusion proteins contain the pectate lyase B (pelB) leader sequence of *Erwinia carotovora* CE^65^. Adding the pelB signal peptide (sp) to the fusion proteins directs them to the Sec translocon for translocation into the periplasm^66^. Suppose Tse5-CT is a soluble protein, the C-terminal of all engineered fusion proteins will localise in the periplasmic space. But, if Tse5-CT is a transmembrane protein, it will contain one or more hydrophobic TM regions. These regions would induce lateral opening of the SecYEG translocase to insert into the cytoplasmic membrane^67,68^. In this scenario, the PhoA-LacZα fusion point could be located in a cytoplasmic, periplasmic or transmembrane region of Tse5-CT, resulting in LacZ, PhoA or mixed activities, respectively. As a control, we generated Tse5-CT-PhoA-LacZα fusion proteins without the signal peptide, and as expected, all the *E. coli* DH5α cells expressing these fusion proteins produce red colonies when plated on dual indicator agar plates (Supplementary Fig. 4a).

*E. coli* DH5α cells expressing the K1229 fusion protein produce blue colonies when plated on dual indicator agar plates, which shows that the C-terminus of the fusion protein localises in the periplasm (Fig. 4e). This result is consistent with the quantification of PhoA-LacZ enzymatic activities in permeabilised *E. coli* DH5α cells expressing the K1229 fusion protein, which indicates the relative PhoA activity (A) is 2.8(6) times that of the relative LacZ activity (Fig. 4f; Table 1: normalised activity ratio [NAR]). Furthermore, the relative PhoA activity of K1229 is the largest of all the engineered fusion proteins.

**Table 1:** Quantification of PhoA-LacZ enzymatic activities in permeabilised *E. coli* DH5α cells expressing Tse5-CT-PhoA-LacZ fusion proteins.

Point of PhoA-LacZ fusion	K1229	A1269	A1281	K1300	Q1317
PhoA^a^ activities (A) (min^−1^)	15(1)	8.8(2)	6.1(3)	8.58(4)	3.3(6)
LacZ^a^ activities (A) (min^−1^)	36(6)	90(7)	85(10)	55(11)	100(2)
PhoA^b^ activities (%) (a.u.)	100(7)	58(1)	40(2)	56.5(2)	21(4)
LacZ^b^ activities (%) (a.u.)	36(6)	89(7)	85(9)	54(11)	100(2)
NAR^c^	2.8(6)	0.65(6)	0.48(7)	1.0(2)	0.22(5)
Localisation	Periplasmic	TM	Cyto/TM?	TM	Cytosolic
Colour	Blue	Purple	Red-purple	Purple	Red

The standard deviations to the last shown digit are represented in parentheses.

^a^The relative LacZ or PhoA enzymatic activities (A) were calculated using Eq. (1).

^b^The relative LacZ or PhoA enzymatic activities (A) were normalised to the maximum enzymatic activity of each enzyme.

^c^The normalised activity ratios (NAR) were calculated using Equation 2 (NAR = (A_PhoA/Highest A_PhoA)/(A_LacZ/Highest A_LacZ)). See the “Methods” section for details.

Interestingly, *E. coli* DH5α cells expressing fusion proteins A1269, A1281 or K1300 produce purple or red-purple colonies (Fig. 4e), consistent with NAR values of 0.65(6), 0.48(7) and 1.0(2), respectively. (Fig. 4f; Table 1). Previous studies using this PhoA-LacZα dual reporter strategy showed that purple and red-purple colonies with NAR values between 2 and 0.5 are obtained when PhoA-LacZα is fused within transmembrane regions (i.e., a combination of both β-galactosidase and phosphatase enzymatic activities due to a mixture of cytoplasmic and periplasmic fusions)^62^. Therefore, the fusion points of A1269 and K1300 fusion proteins are most likely localised in transmembrane regions. Nonetheless, given the borderline NAR value obtained for A1281, it is not possible to readily establish if its fusion point is located in the cytosol or a transmembrane region.

Finally, *E. coli* DH5α cells expressing the full-length spTse5-CT−PhoA-LacZα fusion protein (Q1317) produce red colonies when plated on dual indicator agar plates (Fig. 4e), consistent with having the highest relative LacZ activities of all the engineered fusion proteins (NAR value of ca. 0.22(5); Fig. 4f; Table 1).

### Tse5-CT forms ion-selective membrane pores in vitro

Given that our results support the notion that Tse5-CT is a transmembrane protein that targets the cytoplasmic membrane of intoxicated cells to disrupt their membrane potential, we hypothesised that Tse5-CT could be forming pores that can transport ions across the membrane. This ion transport could be the molecular mechanism employed by Tse5-CT to depolarise intoxicated cells.

To probe its capacity to generate ion channel activity, Tse5-CT dissolved in dimethyl sulfoxide (DMSO) was added to a solution bathing a solvent-free planar phospholipid bilayer (see the “Methods” section for details). Gram-negative bacteria are known to have different chemical compositions in the cytoplasmic and periplasmic space^69,70^. Therefore, to simulate these asymmetric conditions, a salt concentration gradient was used in the membrane chamber, so one side flanking the membrane was kept at 250 mM and the other at 50 mM, both buffered with 5 mM HEPES at pH 7.4.

**Fig. 5:**
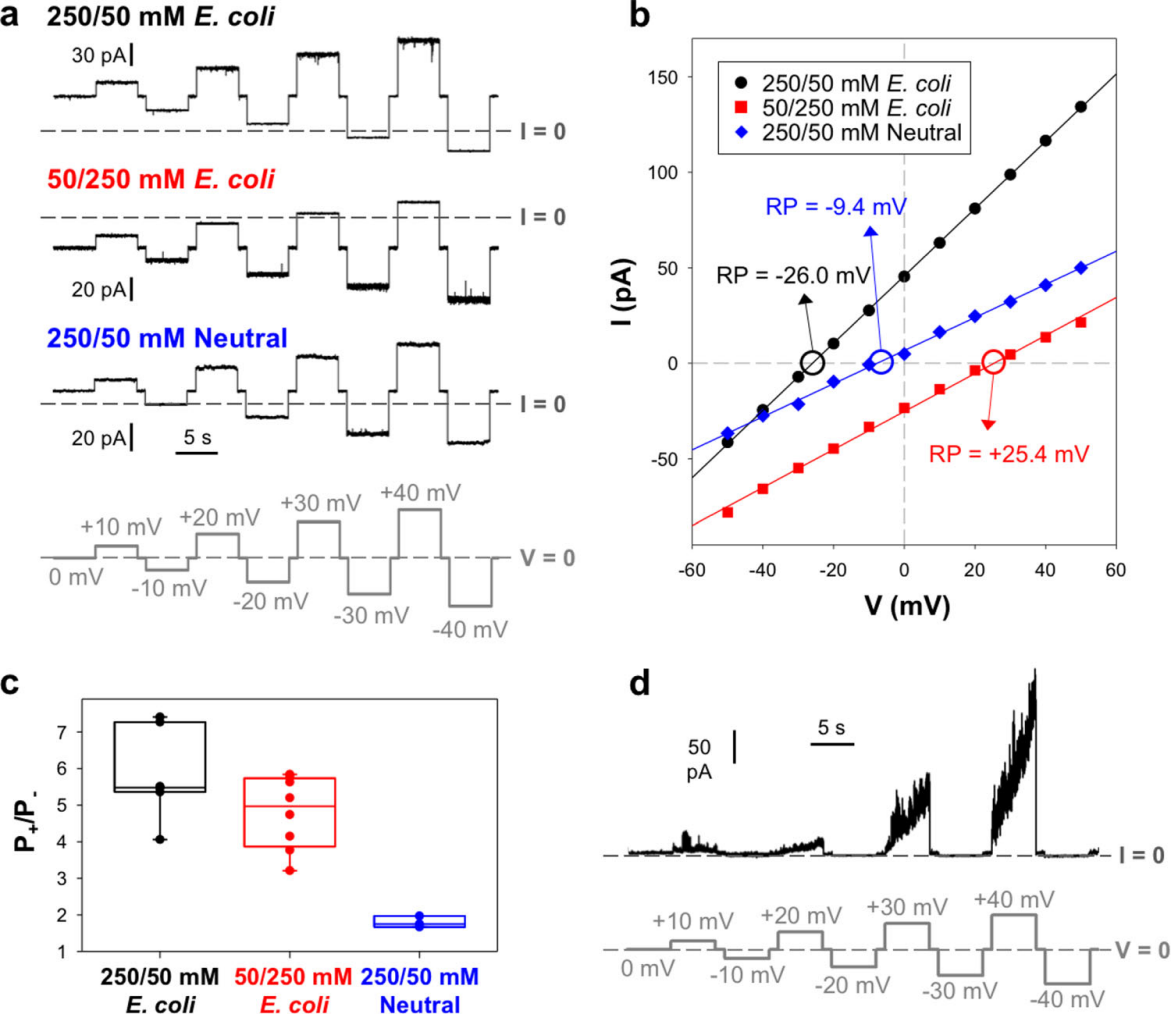
Tse5-CT forms stable pores with ohmic behaviour and preference for cations, and some pores with noisy currents and strong voltage dependence. **a** Representative Tse5-CT-induced stable current traces were obtained in a 250/50 mM (upper panel) or 50/250 mM (middle panel) KCl gradient using a polar lipid extract from *E. coli* to form the membrane. The lower panel shows a representative trace in 250/50 mM KCl gradient when a neutral DOPE membrane was used. The applied voltages are shown at the bottom in light grey. **b** *I*/*V* curves corresponding to the traces shown in (**a**). Linear regressions (solid lines) allow calculation of the conductance (1.76 nS (black), 0.99 nS (red) and 0.95 nS (blue)) and reversal potential (RP, indicated by circles and arrows). A negative (positive) RP corresponds to a cation (anion) selectivity. **c** Permeability ratios, P_K_^+^/P_Cl_^−^, calculated from corresponding reversal potentials using the GHK equation^78^. Solid circles correspond to the individual data points. Data are means of 7 (black), 8 (red), and 4 (blue) independent experiments. **d** Representative Tse5-CT-induced noisy current trace was obtained in a 250/50 mM KCl gradient using the *E. coli* polar lipid extract to form the membrane. The applied voltage is shown at the bottom in light grey. Current records in (**a**) and (**d**) were digitally filtered with 500 Hz using a low-pass 8-pole Bessel filter for better visualisation.

Shortly after Tse5-CT protein was added and the membrane reformed, spontaneous protein insertions were obtained without any applied voltage, revealing ion channel activity with relatively stable currents, as shown in representative traces in Fig. 5a. Tse5-CT-induced currents were obtained using membranes formed with a polar lipid extract from *E. coli* in a 250/50 mM (upper panel) and a 50/250 mM (middle panel) KCl gradient. Protein was always added at the same side of the membrane (*cis*-side), meaning that the gradient direction did not affect the capacity of Tse5-CT to insert into the planar membrane. Control experiments with a neutral bilayer made from 1,2-dioleoyl-*sn*-glycero-3-phosphoethanolamine (DOPE) were also carried out (Fig. 5a, lower panel). In this case, Tse5-CT-induced currents were less frequent and more unstable than with polar membranes.

Figure 5b shows the current–voltage (*I*–*V*) relationships arising from the representative traces shown in Fig. 5a. In all conditions explored, stable currents follow a purely ohmic behaviour, meaning channel conductance (*G* = *I*/*V*) does not change with the applied voltage’s magnitude and polarity. Calculated conductances from all measured *I*–*V* curves vary from 0.6 to 6.6 nS (Supplementary Table 1). The variability of conductances could arise from the insertion of multiple units of the same size or due to variable pore conformations corresponding to different levels of Tse5-CT oligomerization. Discrimination between these two options is out of the scope of the present study. Nonetheless, a *G* of ca. 0.6 nS (the minimal conductive unit obtained, Supplementary Table 1) roughly translates into pores around 1 nm in diameter (see the “Methods” section for details).

The amplitude and frequency of current oscillations in Fig. 5a provide useful information beyond the conductive levels. The power spectral density (PSD) of Tse5-CT-induced currents quantifies the current noise and provides the frequency hallmark^71^ on the primary physical mechanisms responsible for pore formation. Supplementary Fig. 5a shows representative PSDs obtained from Tse5-CT-induced currents. PSDs for all experiments display characteristic 1/f type spectra similar to those found in other proteolipidic pore assemblies^72^. Notably, for all conditions studied, PSDs at low frequencies (5–15 Hz band) follow a parabolic dependence with the applied voltage (Supplementary Fig. 5b). This is a characteristic feature of equilibrium conductance fluctuations^73,74^ and disregards non-equilibrium mechanisms in the pore formation by Tse5-CT (i.e. electroporation^75^).

Experiments under a concentration gradient also allow us to assess the ionic selectivity of the measured pores. Because the mobility of anions and cations is the same in KCl, the sign of the measured current without any applied voltage (the vertical intercept of the *I*–*V* curve in Fig. 5b) provides a hint of the channel preference for anions or cations^76^. In all studied conditions (polar and neutral membrane, 250/50 and 50/250 mM KCl gradients), the sign obtained of the measured currents without applied voltage is consistent with pores displaying cationic selectivity. However, the widely accepted magnitude to quantify selectivity is the so-called reversal potential (RP)^77^, which is the voltage required to yield zero current under a transmembrane gradient (the horizontal intercept of the *I*–*V* curve). Once the measured RP is introduced into the Goldman–Hodgkin–Katz (GHK) equation, we can obtain the permeability ratio P_+_ /P_−_^78^.

Figure 5c shows the permeability ratios (P_+_/P_−_) for different series of experiments. For the 250/50 mM KCl configuration in polar membrane, the measured P_+_/P_−_ = 6.08 ± 1.17 (*N* = 7). This permeability ratio means that the channels have multi-ionic character. Although pores have a marked preference for cations, anions are not excluded and still can permeate through the pore. Reversing the direction of the concentration gradient (50/250 mM KCl) yields a comparable permeability ratio, P_+_/P_−_ = 4.79 ± 0.99 (*N* = 8), and hence similar selectivity to the opposite orientation. Keeping in mind that protein addition always occurs in the *cis* chamber, this implies that Tse5-CT-induced pores are fairly symmetrical structures regarding the charge distribution that regulates ionic selectivity, at least at pH 7.

Taking into consideration that the Tse5-CT net charge is negative and that the *E. coli* lipid extract also contains negatively charged lipids (phosphatidylglycerol (PG) is ~15 wt% of the total and Cardiolipin (CA) ~10 wt%), it is fair to wonder whether Tse5-CT protein participates actively in the pore structure or if it just acts in detergent-like fashion to promote channels formed exclusively by lipids^79^. Control measurements in a neutral membrane with a 250/50 mM KCl gradient yield traces with a positive current without applied voltage (Fig. 5a, lower panel), consistent with cation-selective pores. Quantification through the RP (Fig. 5b) and the corresponding permeability ratio (Fig. 5c) show that pores regulated exclusively by protein charges are still quite selective to cations, with P_+_/P_−_ = 1.70 ± 0.21 (*N* = 4). This cationic selectivity of Tse5-CT-induced pores in neutral membranes demonstrates that Tse5-CT protein forms part of the pore walls.

For the sake of completeness, it should be mentioned that although the majority of traces show pores with relatively stable currents and ohmic *I*–*V* relationships, there is a minor fraction of recordings showing strongly voltage-dependent currents, as shown in Fig. 5d. In these cases, extremely fluctuating currents appear for *V* > 0, increasing rapidly with time, making recording *I*–*V* relationships impossible. In contrast, for *V* < 0 current is almost zero. When *V* = 0, the current is small but measurable and compatible with a structure selective to cations. The successive application of opposite voltage polarities shows that conductive structures do not disappear under *V* < 0, but they just become closed like voltage-gated pores. Interestingly, these voltage-dependent currents that are anecdotic in experiments involving a concentration gradient become much more frequent in experiments performed under symmetric salt concentration conditions.

**Fig. 6:**
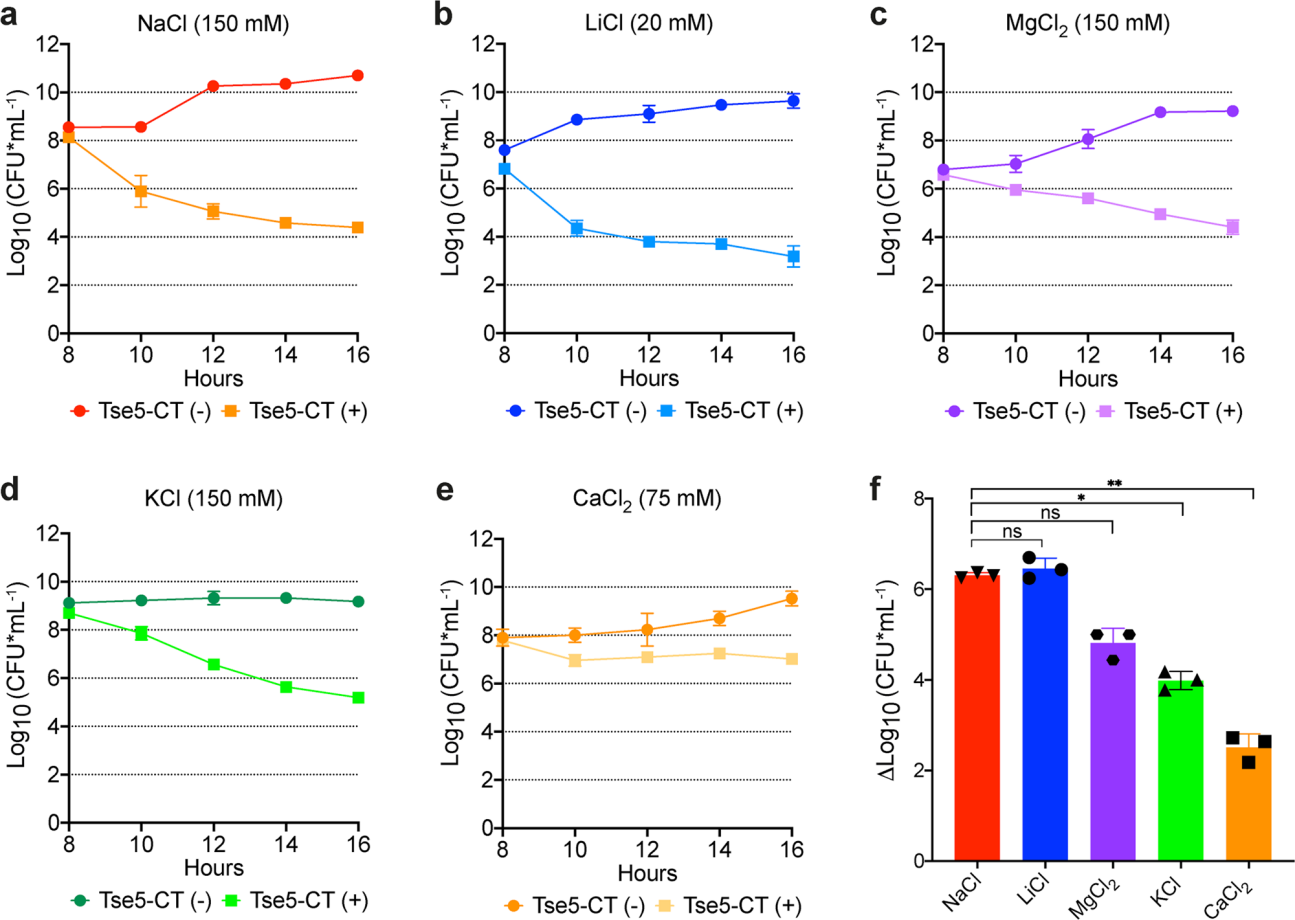
Tse5-CT toxicity is accentuated in the presence of NaCl and LiCl. *P. putida* growth curves expressed in CFU mL^−1^ with and without m-Toluic acid (TA) induction (Tse5-CT+/Tse5-CT−). Each panel shows bacterial growth in liquid medium supplemented with a different salt: 150 mM NaCl (**a**), 20 mM LiCl (**b**), 150 mM MgCl_2_ (**c**), 150 mM KCl (**d**), and 75 mM MgCl_2_ (**e**). The differential growth with and without Tse5-CT expression in each liquid medium is indicated in panel (**f**). Bars show mean ± SD (*n* = 3 independent experiments; ns if *p* > 0,05, * if *p* < 0.05, ** if *p* ≤ 0.01, one-way ANOVA (Brown–Forsythe ANOVA test) with Dunnett´s T3 multiple comparisons test). Some error bars in **a**–**e** are not visible due to overlap with symbols. Source data are provided as a Source Data file.

In addition, to provide some insight on the role of electrolytes in the mechanism of action of Tse5-CT, we evaluated the toxicity of Tse5-CT in *P. putida* growing on liquid media having different salts at sub-inhibitory concentrations (Fig. 6). Within all tested salts, the results indicate that Tse5-CT toxicity is accentuated in the presence of NaCl and LiCl (Fig. 6a, b), resulting in a six-log reduction (Fig. 6f). The lowest Tse5-CT toxicity was observed in the presence of CaCl_2_ (Fig. 6e), resulting in a 2.5-log reduction (Fig. 6f). Whereas intermediate toxicity levels were observed in the presence of MgCl_2_ (Fig. 6c) and KCl (Fig. 6d), resulting in a 4.8- and 4.0-log reduction, respectively (Fig. 6f). The above results are compatible with Tse5-CT-induced membrane pores with cationic selectivity, as described in Fig. 5. However, a connection between the differences in toxicity reported in Fig. 6 and a preferential channel selectivity for particular cations (Na^+^ or Li^+^) is not straightforward for several reasons. On the one side, note that different salts yield notable differences in bacterial growth prior to the activation of Tse5-CT. On the other side, the multi-ionic character of the observed pores in Fig. 5 suggests that ion channel activity of Tse5-CT involves the concerted influx and outflux of cations and anions following the particular ionic gradients developed in the cell growing on liquid media having different salts.

## Discussion

In the current study, we have provided insight into the molecular mechanism that Tse5-CT employs to kill intoxicated bacterial cells. In particular, we show that this molecular mechanism involves the formation of ion-selective membrane pores, which can explain the observed membrane depolarisation of *P. putida* cells expressing the toxin.

In all conditions explored (Fig. 5a), the Tse5-CT-induced currents in lipid membranes follow a purely ohmic behaviour (Fig. 5b). Such voltage-independent conductance has been reported in proteolipidic channels like the protein E of SARS-CoV-1^80,81^ or the classical swine fever virus p7^72^, in total contrast with other channels that show strong voltage-dependent conductance, such as the antibiotic peptide alamethicin^82^, the antimicrobial peptide Syr-E^83^ or melittin peptide from bee venom^84^. Furthermore, the minimal conductive unit obtained (*G* ~ 0.6 nS; Supplementary Table 1) is similar to that of well-known protein channels, like the mitochondrial VDAC^85,86^, which forms 14 Å pores in the mitochondrial outer membrane.

Importantly, the measured permeability ratios (P_+_/P_−_) indicate that Tse5-CT-induced channels have a multi-ionic character (Fig. 5c). Thus, although pores have a marked preference for cations, anions can permeate through the pore. Furthermore, Tse5-CT-induced pores are fairly symmetrical structures, as indicated by similar permeability ratios obtained for 50/250 and 250/50 mM KCl concentration gradients. Nonetheless, this symmetry might not translate in vivo under conditions of acidic stress, where protons unequally titrate each cytoplasmic membrane side. In this scenario, the charge distribution could become asymmetric, and hence the selectivity could depend on the direction of the concentration gradient^87^. Experiments in neutral membranes demonstrate that Tse5-CT protein forms part of the pore walls. This implies that some charged residues in Tse5-CT are necessarily present in the pore walls to account for the measured ionic transport. Tse5-CT contains 13 negatively charged and 19 positively charged residues (Supplementary Fig. 4b). Presumably, some of these negatively charged residues must have an important contribution to the cationic selectivity of Tse5-CT-induced channels, but deciphering their contribution would require determining the molecular structure of the Tse5-CT-induced channels.

In addition, we have observed that Tse5-CT can induce voltage-dependent currents that are anecdotic in experiments involving a concentration gradient but become much more frequent in experiments performed under symmetric salt concentration (Fig. 5d). Based on this result, we could speculate that two different mechanisms of membrane permeabilization could be operating simultaneously. One membrane permeabilization mechanism forms relatively quiet ohmic pores, which in vivo would lead to cell depolarisation. A second membrane permeabilization mechanism that only functions in one voltage polarity and quickly leads to irreproducible membrane disruption, which in vivo would result in cell permeabilization. This channel-pore duality might explain why the expression of Tse5-CT in *P. putida* results in a 46% increase in depolarised cells (Fig. 2b) and a 5% increase in depolarised and permeabilised cells (Fig. 2d). Such behaviour has already been reported in proteolipidic systems and is referred to as *channel-pore dualism*^88^.

Overall, our electrophysiology experiments suggest that Tse5-CT inserts into *E coli* polar membranes yielding proteolipidic pores of unknown architecture (either in the form of barrel-stave, toroidal or arch pores^89^), but in any case, with Tse5-CT located in the pore walls. Furthermore, Tse5-CT-induced pores have an equilibrium nature (are formed spontaneously) and display marked cationic preference although maintaining their multi-ionic character. This multi-ionic character means that changes in cell homoeostasis or cell polarity via Tse5-CT action probably involve the simultaneous transport of several ionic species, which would be consistent given the observed toxicity of Tse5-CT in *P. putida* growing on liquid media having different salts (Fig. 6).

We have observed that the bacteriolytic effect of Tse5-CT on *P. putida* cells is characterised by a rapid and permanent bacterial growth arrest (Fig. 1). This bacteriolytic effect can be correlated with our flow cytometry data, which show that Tse5-CT mainly causes membrane depolarisation (Fig. 2). These experiments were performed in parallel with *P. putida* cells expressing wild-type Tse5-CT and a variant containing the pelB leader sequence (spTse5-CT), which should direct Tse5-CT to the periplasmic space^56^. The results indicate that both proteins (Tse5-CT and spTse5-CT) can depolarise intoxicated cells. Still, the effect is more substantial in the absence of the pelB leader sequence (ca. 46% vs. 21% increase in depolarised cells expressing Tse5-CT or spTse5-CT, respectively). This data is consistent with our electrophysiology experiments that indicate Tse5-CT can spontaneously induce pore formation in lipid membranes regardless of the direction of the KCl gradient. Previous results also indicate that Tse5-CT is toxic when expressed in the cytoplasm of *E. coli*^41^ and when directed to its periplasm^14^. Importantly, we show that the Tse5-CT effect can be reversed by co-expression of the cognate immunity protein Tsi5 (Figs. 1 and 3). This result is in agreement with previous studies showing Tsi5 abrogates Tse5-based intoxication in *P. aeruginosa*^14^ and *E. coli*^54^. Furthermore, using a bacterial competition assay, we observe that Tse5 contributes to the antibacterial activity of *P. aeruginosa* when competing with *P. putida* (see Supplementary Results in Supplementary Information).

Furthermore, using the Langmuir–Blodgett balance, we have demonstrated the capacity of Tse5-CT to partition into the hydrophobic core of a lipid monolayer when introduced from a polar buffer (Fig. 4a, b). This is a substantial result, suggesting that Tse5-CT might contain transmembrane (TM) regions to insert into biological membranes. To evaluate the possibility that Tse5-CT includes TM regions, we engineered a series of Tse5-CT deletion mutants containing the N-terminal pelB leader sequence (sp) and the C-terminal PhoA-LacZα dual reporter protein^62,63^. Evaluation of the enzymatic activity of the dual reporter identified the PhoA-LacZα fusion points located in periplasmic (K1229), cytoplasmic (Q1317), and transmembrane (A1269 and K1300) domains of corresponding chimaeric proteins (Fig. 4e, f; Table 1). This result suggests that Tse5-CT is a transmembrane protein that contains at least one TM region. Furthermore, we show that adding the pelB signal peptide at the N-terminal of Tse5-CT−PhoA-LacZα fusion proteins allows the translocation of the PhoA-LacZα dual reporter into the periplasmic space. However, structural information will be required to decipher the topology of Tse5-CT when spontaneously inserted into target membranes.

Pore-forming toxins (PFTs) are classified into two large groups, α-PFTs and β-PFTs, depending on whether their membrane-spanning domain assembles from α-helices or β-barrels^90^. Whilst structural information will be required to understand the number and arrangement of the transmembrane regions that form the Tse5-CT membrane pore, transmembrane helix predictions suggest a substantial α-helical content with a predicted propensity for membrane insertion (Fig. 4c). Therefore, suggesting Tse5-CT might be an α-PFT.

The membrane potential regulates a wide range of bacterial physiology and behaviours, including pH homoeostasis, membrane transport, motility, antibiotic resistance, cell division, electrical communication, and environmental sensing^91^. Therefore, membrane depolarisation is an excellent strategy to outcompete other bacteria. Based on protein sequence conservation, Tse5-CT seems to be unique to *P. aeruginosa*. Nonetheless, previous pore-forming toxins have been shown to also change the membrane potential of intoxicated cells. These include pore-forming colicins (colicin A^92^, B^93^, E1^94^, Ia^95^, Ib^95^ and N^96^), and some T6SS-effectors (the *P. aeruginosa* Tse4^14^, the *Serratia marcescens* Ssp6^97^, and the *Vibrio cholerae* VasX^98^).

Colicin A, B, E1, Ia, Ib and N cause bacterial depolarisation by forming voltage-gated ion-conducting channels across the plasma membrane of target bacteria. The C-terminal domain of these colicins consists of a tightly packed bundle of 10 α-helices^96,99–102^, that forms a compact water-soluble protein in the producing cell but rearranges itself upon interaction with the inner membrane of the target cell, forming a voltage-gated ion channel^103^. Ssp6 can cause depolarisation of targeted cells without a corresponding increase in permeability of the cytoplasmic membrane and can form ion-selective pores^97^. Similarly, Tse4 disrupted the membrane potential without increased membrane permeability and was suggested to form cation-selective pores^27^. VasX displays some structural homology with pore-forming colicins. It was shown to disrupt the membrane potential with simultaneous permeabilisation of the inner membrane, suggesting that it forms large, non-selective pores, which would cause leakage of ions and other cellular contents^98,104^.

In summary, *P. aeruginosa* produces a plethora of effectors delivered by the T6SS to target essential functions in prey cells. Consequently, these effectors are essential for its pathogenesis, and therefore, deciphering the molecular functions and cellular targets of these toxins could provide unique insight for the next generation of antibiotics. Towards this end, this study shows that Tse5-CT is a transmembrane protein that partitions into the hydrophobic core of model membranes, including lipid monolayers and bilayers. Furthermore, when it inserts in lipid bilayers, our electrophysiology experiments suggest that Tse5-CT assembles proteolipidic pores that display a marked cationic preference although maintaining its multi-ionic character. These findings correlate well with our in vivo results, which indicate that Tse5-CT causes membrane depolarisation and bacterial death. Taken together, these results suggest that Tse5-CT toxicity is produced by the ion-selective pores that it assembles in the cytoplasmic membrane of intoxicated cells.

## Methods

### Growth studies of *P. putida* following Tse5-CT and Tsi5 expression

A detailed list of all strains and plasmids used in this study can be found in Supplementary Table 2. Growth curves were performed in triplicate. In order to evaluate the growth inhibition of Tse5-CT, electrocompetent cells of *Pseudomonas putida* (strain EM383) were transformed with empty plasmid (pS238D1) or plasmids coding for Tse5-CT (pS238D1::*tse5-CT*). To evaluate the protective effect of Tsi5, electrocompetent cells were transformed with plasmids coding for Tse5-CT (pS238D1::*tse5-CT*) and Tsi5 (pSEVA424::*tsi5*). Overnight cultures of transformed *P. putida* EM383 cells were grown in 10 mL Luria-Bertani (LB) and adjusted to OD_600_ = 0.1. Initial cultures were allowed to grow for eight hours (OD_600_ = 1.0−0.6) before inducing the expression of the toxin/immunity proteins. Expression of Tse5-CT and Tsi5 was induced with 1mM m-toluic acid (TA) and 0.1 mM isopropyl 1-thio-β-d-galactopyranoside (IPTG), respectively. Optical density at 600 nm (OD_600_) was measured every hour. After 5 h, half of the cultures were washed with 1 min centrifugation at 9000 × *g*, and the pellet was resuspended in fresh LB broth with appropriate antibiotics. All cultures were grown at 30 °C with agitation (300 rpm) in LB media supplemented with kanamycin (50 μg/mL) (streptomycin at the same concentration in case of bacteria carrying the plasmid pSEVA424:*tsi5*). Every two hours, an aliquot of each bacterial culture was taken and spotted on LB agar plates following serial dilutions to count the number of CFU.

### Flow cytometry studies of *P. putida* cells expressing Tse5-CT, spTse5-CT and Tsi5

See Supplementary Table 2 for a description of strains and plasmids used in this study. To define cell populations, we performed two positive controls, one negative control and a double control (Supplementary Figs. 2 and 3). The negative controls consist of *P. putida* cells transformed with the empty plasmids pSEVA424 and/or pS238D1 and incubated with fluorescent dyes. This assay allows delimiting the healthy population as well as the size and complexity of the cells. For the permeabilisation control, cells were permeabilised by heat shock at 85 °C for 5 min, followed by another 5 min of incubation at 4 °C and then stained with Sytox™ Deep Red. The depolarisation control was obtained by treating cells for 30 min with the antibiotic polymyxin B sulfate (100 µg/mL), followed by 30 min of incubation with DiBAC_4_(3). Finally, a double control was performed by treating cells with heat shock and polymyxin B sulfate and labelling them with DiBAC_4_(3) and Sytox™ Deep Red fluorophores.

In order to evaluate the toxic effect of Tse5-CT and spTse5-CT, electrocompetent cells of *Pseudomonas putida* (strain EM383) were transformed with empty plasmid (pS238D1), or plasmids coding for Tse5-CT (pS238D1::*tse5-CT*) or spTse5-CT (pS238D1::*sptse5-CT*). To evaluate the protective effect of Tsi5, electrocompetent cells were transformed with empty plasmids (pS238D1 and pSEVA424) or plasmids coding for Tse5-CT (pS238D1::*tse5-CT*) and Tsi5 (pSEVA424::*tsi5*). Flasks containing 10 mL of LB media supplemented with kanamycin (50 µg/mL) were inoculated with transformed bacteria, and cell cultures were incubated overnight at 30 °C with agitation (180 rpm). The following day, cultures were diluted to OD_600_ = 0.05 with fresh LB media and led them to grow in the same conditions until they reached the exponential phase (OD_600_ value of ca. 0.5). Tse5-CT and spTse5-CT expressions were induced by adding 1 mM *m*-toluic acid (TA) final concentration. Expression of Tsi5 was induced by adding 0.1 mM IPTG. Ninety minutes after induction, cells were pelleted and suspended in a 1x PBS solution pH 7.4 to a final OD_600_ value of 0.5. Following resuspension, cells were stained with DiBAC_4_(3) (10 µM) and Sytox™ Deep Red (4 µM) and incubated for 30 min under constant shaking in dark conditions. Then, cells were directly analysed in a CytoFLEX cytometer equipped with 488 and 638 nm lasers (Beckman Coulter). Channels used were FITC for DiBAC_4_(3) (Ex_λ_ = 490 nm, Em_λ_ = 516 nm) and APC for Sytox^TM^ Deep Red (Ex_λ_ = 660 nm, Em_λ_ = 682 nm). Data analysis was performed using CytExpert.

### Identification of transmembrane regions in Tse5-CT using PhoA-LacZ dual reporter agar plates

See Supplementary Table 2 for a description of strains and plasmids used in this study. We engineered ten fusion proteins, five with a PelB signal sequence fused to the N-terminal and five without the signal peptide. Fusion proteins contain different fragments of Tse5-CT, corresponding to fragments Tse5-CT_1169–1229_ (K1229), Tse5-CT_1169–1269_ (A1269), Tse5-CT_1169–1281_ (A1281), Tse5-CT_1169–1300_ (K1300), and Tse5-CT_1169–131_ (Q1317; full length Tse5-CT). Chimaeric proteins contain at the C-terminus the PhoA-LacZα dual reporter.

Chemocompetent *E. coli* DH5α cells were transformed by heat shock with plasmids coding for the dual reporter PhoA-LacZ (pKTop parental plasmid), fusion proteins spTse5-CT-PhoA-LacZα, or Tse5-CT-PhoA-LacZα (without the N-terminal PelB signal peptide), followed by bacterial growth in kanamycin (50 µg/mL) selective LB-agar plates. Single colonies were grown overnight in LB media complemented with kanamycin (50 μg/mL). Overnight cultures (20 μL) were spotted onto dual reporter LB-agar platers supplemented with kanamycin (50 μg/mL), Red-Gal (80 μg/mL), X-Pho (100 μg/mL), IPTG (1 mM) and glucose (0.2%). Plates were incubated for 24 h at 37 °C in dark conditions.

### Identification of transmembrane regions in Tse5-CT by measuring PhoA-LacZ enzymatic activity

Single colonies of *E. coli* DH5α cells transformed with pKTop plasmids coding for spTse5-CT-PhoA-LacZα fusion proteins were selected from freshly prepared LB-agar plates supplemented with kanamycin (50 µg/mL) and glucose (0.2%). Cells were grown overnight in LB media supplemented with kanamycin (50 µg/mL) and glucose (0.2%) at 37 °C. Overnight cultures were diluted to an OD_600_ value of 0.1 in fresh LB media, supplemented with kanamycin (50 µg/mL). When cultures reached an OD_600_ = 0.6, the expression of fusion proteins was induced by adding 1 mM IPTG and cell cultures were incubated for 90 min, maintaining the same agitation and temperature conditions before induction.

In order to measure the β-galactosidase (LacZ) activity, 1.2 mL of each bacterial culture was centrifuged for 5 min at 4500 × *g* at room temperature (RT). After removing the supernatant, the cell pellet was resuspended in 1.2 mL of M63 medium (100 mM KH_2_PO_4_, 15 mM (NH4)_2_SO_4_, 1.7 mM FeSO_4_, 1 mM MgSO_4_), and 200 μL were transferred to a 96-well microtiter plate to measure the optical density at 595 nm (OD_595_). In order to permeabilize the cell membranes, the remaining volumes of cell cultures were treated with 100 μL chloroform and 100 μL 0.05% SDS and left for 5 min at 37 °C and 5 min on ice. Once the chloroform settled, 50 µL of the cells-containing upper phase was transferred to the 96-well microtiter plate. Then, 100 μL of the reaction mixture 0.4% ONPG in PM2 medium (70 mM Na_2_HPO_4_, 30 mM NaH_2_PO_4_, 1 mM MgSO_4_, 0.2 mM MnSO_4_) was added to well-containing bacteria, and incubate at RT for 15 min. The reaction was stopped with 50 μL of 1 M Na_2_CO_3_, followed by reading the absorbance of the reaction product at 405 nm (OD_405_).

In order to measure the alkaline Phosphatase (PhoA) activity, 1.2 mL of bacterial cultures were centrifuged for 5 min at 4500 × *g* at RT. After removing the supernatant, cell pellets were resuspended in 1.2 mL of cold wash buffer (10 mM Tris–HCl pH 8.0, 10 mM MgSO_4_) to remove phosphate ions from the medium. The wash buffer was removed by 5 min of RT centrifugation at 4500 × *g* and resuspension of the cell pellet in 1.2 mL of cold PM1 medium (1 M Tris–HCl, pH 8.0, 0.1 mM ZnCl_2_, 1 mM iodoacetamide). From resuspended cells in PM1 medium, 200 μL were transferred to a 96-well microtiter plate to measure the optical density (OD_595_). Membrane permeabilization was performed with the remaining cell cultures as previously described. Following permeabilization, 50 µL of the cells-containing upper phase was transferred to the 96-well microtiter plate. Then, 100 μL of the reaction mixture 0.4% *p*-NPP in 1 M Tris–HCl, pH 8.0, was added to well-containing bacteria and incubated at RT for 60 min. The reaction was stopped by adding 50 μL of 2 M NaOH, followed by reading the absorbance of the reaction product at 405 nm (OD_405_).

The relative enzymatic activity of β-galactosidase (LacZ) or alkaline phosphatase (PhoA) (*A*) was calculated using Eq. (1), which considers the optical density (OD_595_) of the sample and the absorbance of the reaction products (OD_405_)

1 $${A}_{{{\rm {LacZ}}}{{\rm {or}}}{{\rm {PhoA}}}}=1000\,{\cdot }\,\left(\frac{{{{\rm {OD}}}}_{405}{{\rm {sample}}}-{{{\rm {OD}}}}_{405}{{\rm {control}}\; {{{{{\rm{well}}}}}}}}{{{{\rm {OD}}}}_{595}\,{{\rm {sample}-{{OD}}}}_{595}\,{{\rm {control}}\,{{{{{\rm{well}}}}}}}}\right)/t\left({min }\right){{\rm {of}}\; {{{{{\rm{incubation}}}}}}}$$

After obtaining the relative enzymatic activity for each Tse5-CT-PhoA-LacZα fusion protein, the normalised activity ratio (NAR) is calculated (Eq. (2)). Each measured activity is normalised by the highest relative enzymatic activity (A) of the series.

2 $${{\rm {NAR}}}=\frac{\left({A}_{{{\rm {PhoA}}}}/{{{\rm {Highest}}}A}_{{{\rm {PhoA}}}}\right)}{\left({A}_{{{\rm {LacZ}}}}/{{{\rm {Highest}}}A}_{{{\rm {LacZ}}}}\right)}$$

### Expression of Tse5 and purification of Tse5-CT to study insertion in lipid monolayers and bilayers

The Tse5-CT toxin was obtained as a self-cleavage product of the full-length protein Tse5 (Supplementary Fig. 4b–f). The *Pseudomonas aeruginosa* tse5 gene (PA2684) was synthesised by GenScript (GenScript, NJ, USA). The construct contains a 5′ extension encoding for a 9xHis tag and a tobacco etch virus protease cleavage site (ATGGGCAGCAGCCATCATCATCATCATCATCATCATCACAGCAGCGGCGAAAACCTGTATTTTCAGGGCGGATCC). The construct was cloned into a pET29a(+) vector between the NdeI and HindIII restriction sites (pET29a(+)::*9xhis-Tse5*). This construct codes for the protein sequence shown in Supplementary Fig. 4b.

For the expression of Tse5 protein, *Escherichia coli* Lemo21(DE3) cells were transformed with the pET29a::*his-tag-tse5* plasmid and grown in LB agar medium supplemented with 50 µg/mL kanamycin, 34 µg/mL chloramphenicol and 2 mM rhamnose at 37 °C^105^. For protein overexpression, rhamnose was removed from the LB medium. When cells reached an OD_600_ value of ca. 0.7, Tse5 expression was induced by adding to the culture isopropyl β-d-1-thiogalactopyranoside (IPTG) at the final concentration of 1 mM, and the temperature was dropped to 18 °C. After ca. 18 h, the cells were harvested and frozen for later use.

Cell pellet obtained from 4 L culture was resuspended in 60 mL of 50 mM Tris–HCl pH 8.0, 500 mM NaCl, 20 mM imidazole and 4 μL of benzonase endonuclease and 1 tablet of protease inhibitor cocktail (cOmplete, EDTA-free, Roche). Cells were then disrupted by sonication, and the suspension was centrifuged for 40 min at 43,000 × *g*. The supernatant was filtered with a 0.2 μm syringe filter and subjected to immobilised metal affinity chromatography using a HisTrap HP column of 5 mL (GE Healthcare) on a fast protein liquid chromatography system (ÄKTA FPLC; GE Healthcare) equilibrated with 25 mL of 50 mM Tris–HCl pH 8, 500 mM NaCl and 20 mM imidazole (solution A). The column was washed with solution A at 0.5 mL/min until no change in absorbance at 280 nm was detected. Elution was performed with a linear gradient between 0% and 50% of 50 mM Tris–HCl pH 8, 500 mM NaCl and 500 mM imidazole (solution B) in 40 mL and 2 mL/min. Fractions containing Tse5 protein were pooled, and protein concentration was estimated by measuring absorbance at 280 nm. Tse5 protein was injected into a HiLoad Superdex 200 26/600 pg, previously equilibrated with 20 mM Tris–HCl pH 8, 150 mM NaCl and 2 mM DTT. Tse5 eluted as a single monodispersed peak (Supplementary Fig. 4c), but SDS–PAGE revealed three protein fragments (Supplementary Fig. 4d). The protein was then concentrated using Amicon centrifugal filter units of 30 kDa molecular mass cut-off (Millipore) to a final concentration of ca. 15 mg mL^−1^ (ca. yield: 6 mg/L). The concentrated protein sample was diluted in solution A containing 8 M urea to a final concentration of 6 M urea to denature Tse5 and separate the three fragments. To remove the 9xHis-tag-containing N-terminal fragment, the denatured protein solution was added to a nickel resin (GE Healthcare) previously washed and equilibrated with buffer A with 8 M urea and left for 5 min at 4 °C under agitation. Following centrifugation of the nickel resin at 15,000×*g*, the unbound fraction containing the Tse5-CT and the central Rhs fragments were recovered. Tse5-CT was separated from the Rhs fragment by differential precipitation with ammonium sulfate^106^. First, the Rhs fragment was precipitated at 0.9 M ammonium sulfate, followed by 15 min centrifugation at 15,000×*g*. The supernatant containing Tse5-CT was subsequently precipitated with 3 M ammonium sulfate. Precipitated Tse5-CT was washed with MiliQ water, flash-frozen in liquid nitrogen, and lyophilised. Once lyophilised, samples were stored at −20 °C, ready to use. The purity and identity of the protein were verified by SDS–PAGE and mass spectrometry (Supplementary Fig. 4d, e and Supplementary Note 1). The N-terminal sequencing of purified Tse5-CT indicates that the Ile1169 residue corresponds to the N-terminus of the protein (Supplementary Fig. 4d and Supplementary Note 2).

### Residues D1141 and D1164 are essential for the auto-cleavage of Tse5-CT

GenScript (GenScript, NJ, USA) derived plasmids pET29a(+)::*D1141A* and pET29a(+)::*D1164A* from the parental plasmid pET29a(+)::*9xhis-Tse5* (see the “Methods” section and Supplementary Table 2 for details). The two new plasmids code for single-point mutations at residues D1141 and D1164 in Tse5, respectively, which were mutated to alanine. Expression in *E. coli* Lemo21 cells and purification by FLPC of point-mutants D1141A and D1164A were carried out following the protocol derived for 9xhis-Tse5. Protein purity was evaluated by SDS–PAGE, which confirmed both mutants are unable to cleave the Tse5-CT (Supplementary Fig. 4f).

### Study of Tse5-CT partitioning in lipid monolayers

The capacity of Tse5-CT to penetrate into lipid monolayers was assessed by measuring its maximum insertion pressure (MIP) using the Langmuir–Blodgett balance technique with a DeltaPi-4 Kibron tensiometer (Helsinki, Finland). Each experiment was performed in a fixed-area circular trough (Kibron μTrough S system, Helsinki, Finland) of 2 cm in diameter, where 1.25 mL of the aqueous phase was added (5 mM Hepes pH 7.4, 150 mM NaCl). The temperature of the Langmuir balance was controlled thermostatically by a water bath at 25 °C (JULABO F12). The monolayer was formed by spreading over the aqueous surface *E. coli* total lipid extract (Avanti Polar lipids) dissolved in chloroform at 1 mg/mL with a Hamilton microsyringe until the desired initial monolayer surface pressure was reached (Π_0_). Experiments at different initial surface pressure (Π_0_) values were recorded by changing the amount of lipid applied to the air-water interface (Π_0_ value ranging from 15 to 30 mN/m). Then Tse5-CT dissolved in DMSO was injected into the aqueous subphase to facilitate incorporation into lipid monolayers (final concentration of 0.4 μM) while controls were carried out by injecting DMSO alone. Changes in surface pressure were monitored over time and were plotted as a function of Π_0_. These data were fitted to a linear regression model, and the maximum insertion pressure was determined by extrapolation (*y* value when *x* = 0).

### Study of Tse5-CT pore-forming activity in planar lipid bilayers

Planar lipid membranes were formed by using a solvent-free modified Montal–Mueller technique^107^. Lipid was prepared from chloroform solutions of either a natural polar extract from *E. coli* or pure dioleoyl-phosphatidylethanolamine (DOPE). All lipids were purchased from Avanti Polar Lipids (Alabaster, AL). *E. coli* polar lipid extract headgroup composition is 67% phosphatidylethanolamine, 23.2% phosphatidylglycerol, and 9.8% cardiolipin (Avanti Polar Lipids). Acyl chains are the mixture naturally present in *E. coli*^108^. All lipids were dissolved in pentane at a 5 mg/mL concentration after chloroform evaporation. Two monolayers were made by adding 10–30 µL of the lipid solution to each compartment of a Teflon chamber (so-called *cis* and *trans*), each filled with 1.8 mL salt solutions. The two compartments were partitioned by a 15 μm-thick Teflon film with a ca. 100 μm diameter orifice where the bilayer formed. The orifice was pre-treated with a 3% solution of hexadecane in pentane. After pentane evaporation, the level of solutions in each compartment was raised above the orifice so the planar bilayer could form by apposition of the two monolayers. Capacitance measurements monitored correct bilayer formation. After bilayer formation, Tse5-CT dissolved in DMSO was added to the *cis* compartment.

To carry out the electrical measurements, an electric potential was applied using in-house prepared Ag/AgCl electrodes in 2 M KCl, 1.5% agarose bridges assembled within standard 250 μL pipette tips. Potential is defined as positive when it is higher at the side of the protein addition (the *cis* side) while the *trans* side is set to ground. An Axopatch 200B amplifier (Molecular Devices, Sunnyvale, CA) in the voltage-clamp mode was used for measuring the current and applying potential. Data were filtered by an integrated low-pass 8-pole Bessel filter at 10 kHz, saved with a sampling frequency of 50 kHz with a Digidata 1440A (Molecular Devices, Sunnyvale, CA), and analysed using pClamp 10 software (Molecular Devices, Sunnyvale, CA). The membrane chamber and the head stage were isolated from external noise sources with a double metal screen (Amuneal Manufacturing Corp., Philadelphia, PA). The described set-up can resolve currents of the order of picoamperes with a time resolution below the millisecond.

Current measurements were performed with a concentration gradient of either 250 mM KCl *cis*/50 mM KCl *trans* (250/50 mM) or 50 mM KCl *cis*/250 mM KCl *trans* (50/250 mM) or using 150 mM KCl symmetrical solutions. All solutions were buffered with 5 mM HEPES at pH 7.4. The pH was adjusted by adding HCl or KOH and controlled during the experiments with a GLP22 pH meter (Crison). Steady current at each applied potential was calculated from a single Gaussian fitting of histograms of current values. Conductance (*G* = *I*/*V*) was obtained from the slope of the calculated current–voltage (*I*–*V*) curves.

Selectivity measurements were performed in experiments with a concentration gradient of 250/50 or 50/250 mM. Once the protein was inserted, a net ionic current appeared due to the existence of one or several selective pores under a salt concentration gradient. Selectivity was quantified by measuring the reversal potential (RP), which is the applied voltage needed to cancel the current. If the channel is neutral, RP equals zero, while it becomes non-zero when the channel is selective to anions or cations. When the concentration gradient is 250/50 mM, a negative RP corresponds to cation-selective channels; with the opposite gradient (50/250 mM), a positive RP indicates cationic selectivity. RP was obtained from either the linear regression of the measured IV curves or by manually cancelling the observed current. All RP values were corrected by the liquid junction potential from Henderson’s equation to eliminate the contribution of the electrode’s agarose bridges^76^. As a first approximation, the pore conductance can be written as *G* ~ *κπr*^2^/*L*, where *κ* is the electrolyte conductivity (*κ* ~ 1.8 S/m for 150 mM KCl, the average concentration in our reversal potential experiments) and *L* the pore length (the lipid bilayer is about 4 nm in length). This allows for a rough estimation of the characteristic pore radius that would be r ~ 0.65 nm for *G* ~ 600 pS.

To carry out the current fluctuation analysis, the power spectral density (PSD) of the current fluctuations was obtained directly from the measured current traces with the pClamp 10 software (Molecular Devices, LLC.). The PSD generates a frequency-domain representation of the time-domain data, revealing the power levels of the different frequency components present in the signal and allowing the rationalisation of physical mechanisms that are difficult to identify directly from current measurements^71^. PSD was measured by calculating the Fast Fourier Transform from the digitised signal with a spectral resolution of 0.76 Hz. For each signal, the available spectral segments were averaged with 50% overlap. To evaluate the increase of the PSD amplitude with the measured current, PSDs were averaged in 5–15 Hz. The increase of the PSD amplitude as the square of the average current is a signature of conductance fluctuations^109,110^.

### Evaluation of Tse5-CT toxicity in *P. putida* growing on liquid media with different salts

Overnight cultures of *P. putida* EM383 cells transformed with pS238D1::*tse5-CT* were grown in 10 mL of Luria-Bertani (LB) medium without salt (LBNS: 10 g/L tryptone, 5 g/L yeast extract) supplemented with kanamycin (50 μg/mL). Initial cultures were adjusted to OD_600_ = 0.1 in new LBNS supplemented with different salts (150 mM NaCl/20 mM LiCl/150 mM MgCl_2_/150 mM KCl/75 mM CaCl_2_) and kanamycin. Cultures were allowed to grow to an exponential phase (OD_600_ = 0.6–0.9) before inducing toxin expression with 1 mM m-Toluic acid (TA). Optical density at 600 nm (OD_600_) was measured every hour. Samples of *P. putida* cells were taken every 2 h, and dilutions were spotted on LB agar plates. Colony-forming units (CFU) were counted 24 h later.

### Statistics and reproducibility

Statistical analyses were performed using GraphPad Prism v.9 and are detailed in the figure legends.

### Reporting summary

Further information on research design is available in the Nature Research Reporting Summary linked to this article.

## Supplementary information

Supplementary Information

Description of Additional Supplementary Files

Supplementary Data 1

Reporting Summary

## Data Availability

The authors declare that source data supporting the findings of this study are available in the Flow Repository database (https://flowrepository.org/) under accession ID FR-FCM-Z5R4, or within the paper and its Supplementary Information files. Supplementary Data 1 is available as an additional supplementary file and contains numeric data for charts included within the article. Supplementary Notes 1 and 2 are available in the Supplementary Information file and contain the LC–ESI–MS report and the N-terminal sequencing report for Tse5-CT. Plasmids and their corresponding sequences can be obtained from Addgene under accession IDs: 192948–192964. Uncropped and unedited gel images are included in Supplementary Fig. 6.
