# Supplementary material for: The P. aeruginosa effector Tse5 forms membrane pores disrupting the membrane potential of intoxicated bacteria
Source: Commun Biol. 2022 Nov 5;5:1189. doi: 10.1038/s42003-022-04140-y (PMC9637101; doi:10.1038/s42003-022-04140-y)
Supplement: Supplementary file 1 — Supplementary Information [file 42003_2022_4140_MOESM1_ESM.pdf]

SUPPLEMENTARY INFORMATION

The *P. aeruginosa* effector Tse5 forms membrane pores disrupting the membrane potential of intoxicated bacteria

Amaia González-Magaña[1,#], Jon Altuna[1,#], María Queralt-Martín[2], Eneko Largo[1,3], Carmen Velázquez[1], Itxaso Montánchez[3], Patricia Bernal[4], Antonio Alcaraz[2], David Albesa-Jové[1,5*]

[1] Instituto Biofisika (CSIC, UPV/EHU), Fundación Biofísica Bizkaia/Biofisika Bizkaia Fundazioa (FBB) and Departamento de Bioquímica y Biología Molecular, University of the Basque Country, 48940 Leioa, Spain.

[2] Laboratory of Molecular Biophysics, Department of Physics, University Jaume I, 12071 Castellón, Spain

[3] Departamento de Inmunología, Microbiología y Parasitología, University of the Basque Country, 48940 Leioa, Spain.

[4] Departamento de Microbiología, Facultad de Biología, Universidad de Sevilla, 41012 Sevilla, Spain

[5] Ikerbasque, Basque Foundation for Science, 48013 Bilbao, Spain

[#] These authors contributed equally to this work

Running title: Tse5 forms ion-selective pores to depolarise intoxicated bacterial cells

* To whom correspondence should be addressed: David Albesa-Jové, Instituto Biofisika (UPV/EHU, CSIC), Scientific Park of the University of the Basque Country, Leioa, E-48940, Spain; Phone: +34 94 601 5171; E-mail: david.albesa@ehu.eus

TABLE OF CONTENTS

Supplementary Results 3

Tse5 contributes to the antibacterial activity of P. aeruginosa when competing againts P. putida 3

Supplementary Tables 4

Supplementary Table 1. Conductances obtained from I-V curves 4

Supplementary Table 2. Strains and Plasmids used in this study 4

Supplementary Figures 7

Supplementary Note 1 LC-ESI-MS report for Tse5-CT 14

Supplementary Note 2 N-terminal sequencing report 18

Supplementary references 26

Supplementary Results

Tse5 contributes to the antibacterial activity of *P. aeruginosa* when competing againts *P. putida*

In vitro competition assays were performed on LB Lennox (5 g/L NaCl) agar (1.5% w/v) plates, as previously described[1]. Briefly, overnight bacterial cultures were washed and adjusted to an OD_{600} of 10 in sterile PBS and mixed in a 1:1 ratio (*P. aeruginosa* (attacker) : *P. putida* (prey)). To differentiate the antibacterial activity of Tse5 from other T6SS effectors, we employed three *P. aeruginosa* knockouts: *P. aeruginosa* Δ*retS*, Δ*retS* Δ*vgrG1*, and Δ*retS* Δ*tse5*. The *P. aeruginosa* : *P. putida* mixtures were grown on LB agar plates at 30 °C for 5 hours and then collected using an inoculating loop and resuspended in sterile PBS. The outcome of the competition was quantified by counting colony forming units (CFUs) using antibiotic selection at 5 hours. The prey strain *P. putida* KT2440R harbours a mini-Tn7 transposon[2] inserted at the *attTn7* site on the chromosome that confers resistance to gentamicin and was used for antibiotic selection. Seventeen biologically independent experiments were performed.

The bacterial competition assays show the antibacterial activity of *P. aeruginosa* effectors secreted by the T6SS (Supplementary Fig. 1). *P. putida* survival increases by 0.45 logs when VgrG1-dependent effectors are not secreted (Δ*retS* Δ*vgrG1*), while survival increases 0.25 logs when Tse5 is not present (Δ*retS* Δ*tse5*). Although these survival increments are small, they are statistically significant, providing experimental evidences of the Tse5 antibacterial activity during bacterial competition.

Supplementary Tables

Supplementary Table 1. Conductances obtained from I-V curves

E. coli 250/50 mM	*E. coli* 50/250 mM	Neutral 250/50 mM
0.57 nS<br>0.57 nS<br>1.54 nS<br>1.76 nS<br>1.78 nS<br>1.83 nS	0.99 nS<br>1.10 nS<br>2.22 nS<br>4.49 nS	0.95 nS<br>6.62 nS

Supplementary Table 2. Strains and Plasmids used in this study

Strain	Relevant characteristics	Use	Origin
E. coli			
**DH5α**	*F - endA1 glnV44 thi-1 recA1 relA1 gyrA96 deoR nupG purB20 φ80dlacZ ΔM15 Δ(lacZYA- argF)*U169, hsdR17 (r K–mK+), λ-	*In vivo* study of Tse5-CT TM regions using PhoA-LacZα dual reporter	Invitrogen
**Lemo21**	BL21(DE3) strain with an extra plasmid harbouring the gene encoding T7 lysozyme, an inhibitor of the T7 RNAP, under control of the well-titratable rhamnose promoter	Heterologous expression of Tse5 for purification of Tse5-CT	[3]
P. putida			
**EM383**	mt-2 derivative cured of the TOL plasmid pWW0 Δprophage1 Δprophage4 Δprophage3 Δprophage2 ΔTn7 ΔendA-1 ΔendA-2 ΔhsdRMS Δflagellum, ΔTn4652 ΔrecA	Flow cytometry and growth inhibition studies	[4]
**KT2440R**	Harbours a mini-Tn7 transposon inserted at the *attTn7* site on the chromosome that confers resistance to gentamicin	It was used for antibiotic selection in the bacterial competition assay	This work
P. aeruginosa			
**PAO1 Δ*retS***	Knockout mutant with activated H1-T6SS	It was used as attacker in the bacterial competition assay	[5]
**PAO1 Δ*retS* Δ*vgrG1***	Knockout mutant with activated H1-T6SS and deleted *vgrG1* gene	It was used as attacker in the bacterial competition assay	[5]

**PAO1 Δ*retS* Δ*tse5***	Knockout mutant with activated H1-T6SS and deleted *tse5* gene	It was used as attacker in the bacterial competition assay	[5]
**Plasmid**	**Relevant characteristics**	**Use**	**Origin**
**pKTop**	A vector expressing the reporter protein $PhoA_{22-472}$ / $LacZ_{4-50}$, p15 ori; Km^r	Parental vector	[6]
**pKTop::*sptse5-CT*$_{1169-1229}$**	Derived from pKTop expressing the Tse5-$CT_{1169-1229}$ / $PhoA_{22-472}$ / $LacZ_{4-60}$ fusion protein **with the PelB** signal peptide fused at the N-terminus	Membrane insertion study	This work
**pKTop::*sptse5-CT*$_{1169-1269}$**	Derived from pKTop expressing the Tse5-$CT_{1169-1269}$ / $PhoA_{22-472}$ / $LacZ_{4-60}$ fusion protein **with the PelB** signal peptide fused at the N-terminus	Membrane insertion study	This work
**pKTop::*sptse5-CT*$_{1169-1281}$**	Derived from pKTop expressing the Tse5-$CT_{1169-1281}$ / $PhoA_{22-472}$ / $LacZ_{4-60}$ fusion protein **with the PelB** signal peptide fused at the N-terminus	Membrane insertion study	This work
**pKTop::*sptse5-CT*$_{1169-1300}$**	Derived from pKTop expressing the Tse5-$CT_{1169-1300}$ / $PhoA_{22-472}$ / $LacZ_{4-60}$ fusion protein **with the PelB** signal peptide fused at the N-terminus	Membrane insertion study	This work
**pKTop::*sptse5-CT*$_{1169-1317}$**	Derived from pKTop expressing the Tse5-$CT_{1169-1317}$ / $PhoA_{22-472}$ / $LacZ_{4-60}$ fusion protein **with the PelB** signal peptide fused at the N-terminus	Membrane insertion study	This work
**pKTop::*tse5-CT*$_{1169-1229}$**	Derived from pKTop expressing the Tse5-$CT_{1169-1229}$ / $PhoA_{22-472}$ / $LacZ_{4-60}$ fusion protein **without PelB** signal peptide	Membrane insertion study	This work
**pKTop::*tse5-CT*$_{1169-1269}$**	Derived from pKTop expressing the Tse5-$CT_{1169-1269}$ / $PhoA_{22-472}$ / $LacZ_{4-60}$ fusion protein **without the PelB** signal peptide	Membrane insertion study	This work
**pKTop::*tse5-CT*$_{1169-1281}$**	Derived from pKTop expressing the Tse5-$CT_{1169-1281}$ / $PhoA_{22-472}$ / $LacZ_{4-60}$ fusion protein **without the PelB** signal peptide	Membrane insertion study	This work
**pKTop::*tse5-CT*$_{1169-1300}$**	Derived from pKTop expressing the Tse5-$CT_{1169-1300}$ / $PhoA_{22-472}$ / $LacZ_{4-60}$ fusion protein **without the PelB** signal peptide	Membrane insertion study	This work
**pKTop::*tse5-CT*$_{1169-1317}$**	Derived from pKTop expressing the Tse5-$CT_{1169-1317}$ / $PhoA_{22-472}$ / $LacZ_{4-60}$ fusion protein **without the PelB** signal peptide	Membrane insertion study	This work
**pS238D1**	Vector optimized to express toxins in *P. putida* with transcriptional repression, pBBR1 oriV and oriT; Km^r	Parental vector	[7]
**pS238D1::*tse5-CT***	Derived from pS238D1 that expresses the protein Tse5-$CT_{1169-1317}$	Biological function study	This work

**pS238D1::*sptse5-CT***	Derived from pS238D1 that expresses the Tse5-$CT_{1169-1317}$ protein with the PelB signal peptide fused at the N-terminus	Biological function study	This work
**pSEVA424**	Vector for protein expression in a broad-spectrum of organisms, oriV and oriT; Str^r	Parental vector	[8]
**pSEVA424::*tsi5***	Derived from $pSEVA_{424}$ that expresses the protein $Tsi5_{1-76}$	Biological function study	This work
**pS238D1::*sptse5-CT_phoA-lacZα***	The gene coding for spTse5-$CT_{1169-1317}$-$PhoA_{22-474}$-$LacZ_{4-60}$ fusion protein was subcloned into the pS238D1 plasmid.	Test the biological function of the spTse5-$CT_{1169-1317}$-$PhoA_{22-474}$-$LacZ_{4-60}$ fusion protein in *P. putida*	This work
**pET29a(+)::*9xhis-Tse5***	Plasmid harbouring a construct based on tse5 and coding for a 9xHis tag and a tobacco etch virus protease cleavage site at the 5' end.	Heterologous expression of Tse5 in *E. coli* Lemo21 cells for purification of Tse5-CT	This work
**pET29a(+)::*D1141A***	Plasmid derived from pET29a(+)::*9xhis-Tse5* coding for a D1141A point mutation.	Test the activity of the putative aspartyl protease motif DPXGL-(18)-DPXGL	This work
**pET29a(+)::*D1164A***	Plasmid derived from pET29a(+)::*9xhis-Tse5* coding for a D1164A point mutation.	Test the activity of the putative aspartyl protease motif DPXGL-(18)-DPXGL	This work

Supplementary Figures

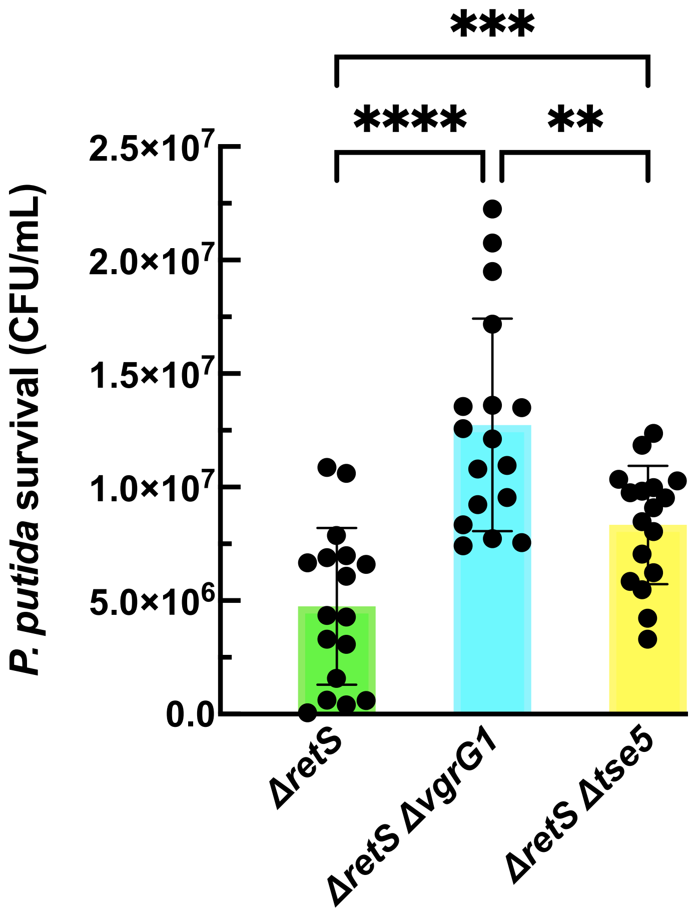

Supplementary Fig. 1 | Competition assays between *P. aeruginosa* PAO1 *retS* attacker strains and *P. putida* KT2440R prey strain. The prey strain harbors a mini-Tn7 transposon inserted on the chromosome that confers gentamycin resistance. The *P. aeruginosa* PAO1 *retS* (parental strain) and its isogenic *vgrG1* and *tse5* mutants were co-incubated with *P. putida* KT2440R for 5 hours. Colony-forming unit (CFU) quantifications of the prey survival after competition were performed on gentamycin selection. Graph shows means ±SD, significance is indicated by ** if $p < 0.01$, *** if $p < 0.001$ or **** if $p < 0.0001$. Statistical analysis was performed using RM one-way ANOVA multiple comparison (n=17).

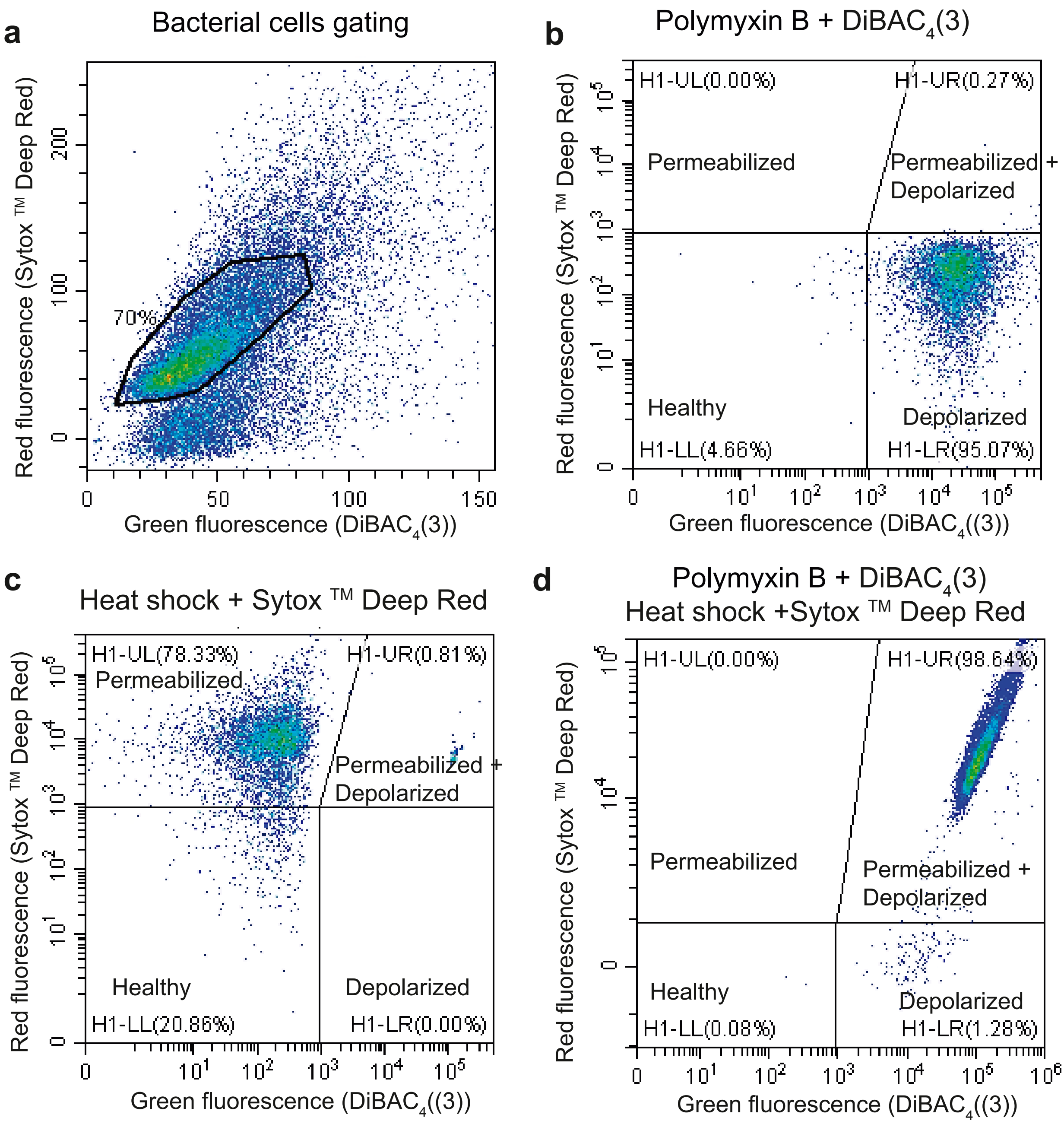

Supplementary Fig. 2 | Analysis of bacterial control cells by Flow Cytometry a. *P. putida* cells gating to classify cell size and complexity, with the X-axis being the size (FSC channel) and the Y-axis is the complexity (SSC channel). **b**. Not treated cells to identify healthy population. **c**. Cells treated with polymyxin B (100 µg/ml) to classify the depolarized population. **d**. Cells treated with heat shock to classify the permeabilized population. **e**. Cells treated with polymyxin B (100 µg/ml) and heat shock to identify depolarized and permeabilized population. Red fluorescence emitted by the Sytox™ Deep Red fluorescent marker (APC channel), a cell permeability marker, is represented on the X-axis, while the green fluorescence obtained by the $DiBAC_4(3)$ marker (FITC channel), a fluorophore sensitive to membrane depolarization.

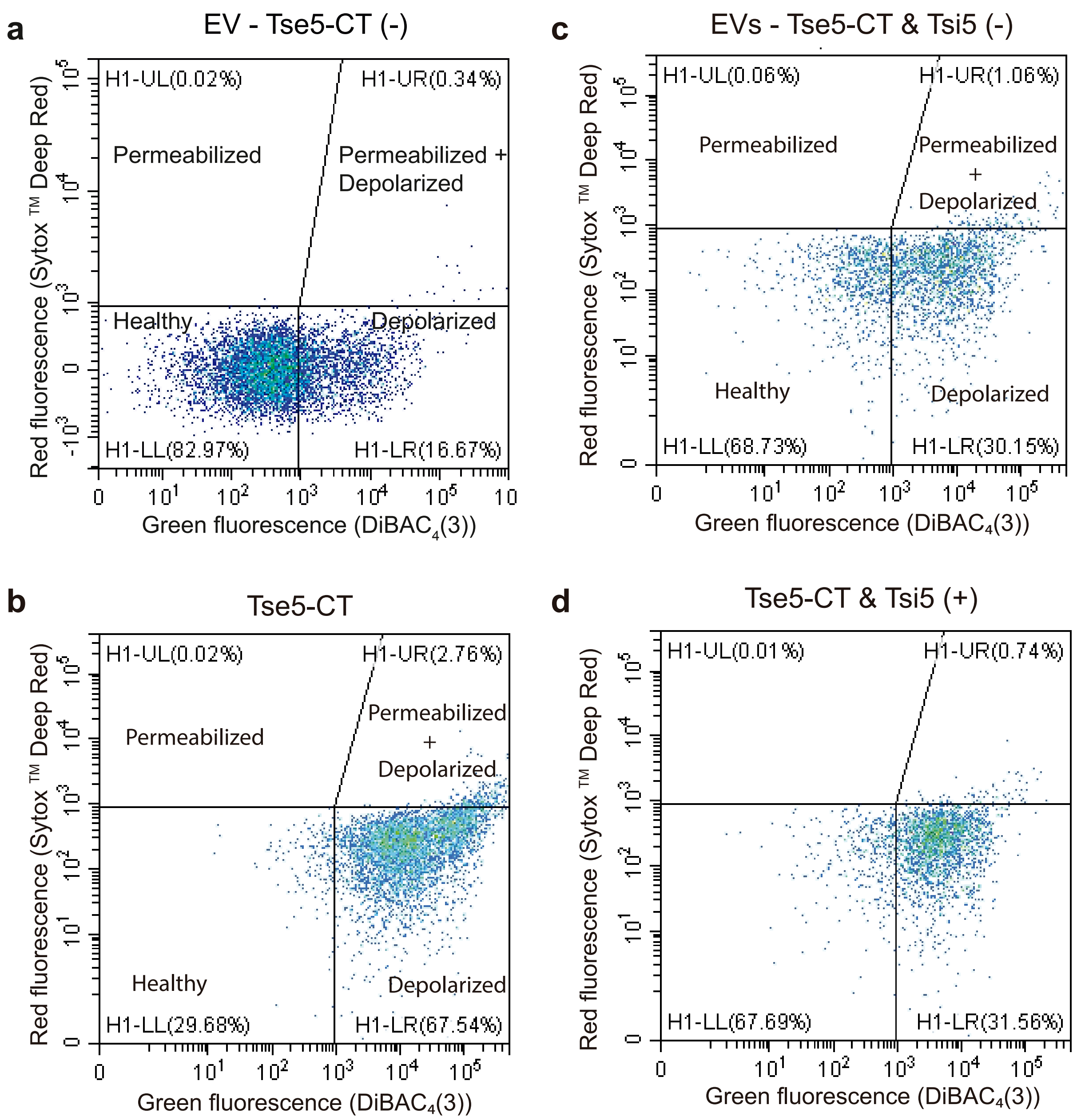

Supplementary Fig. 3 | Bacterial cell analysis by Flow Cytometry reveal Tse5-CT causes membrane depolarization, and Tsi5 can protect from Tse5-induced effect. a. Empty vector carrying cells to identify basally permeabilized or/and depolarized cell populations. **b**. *P. putida* cells expressing Tse5-CT. **c**. *P. putida* cells expressing spTse5-CT, which translocate to the periplasm through the SEC pathway. **d**. *P. putida* cells expressing Tse5-CT and Tsi5.

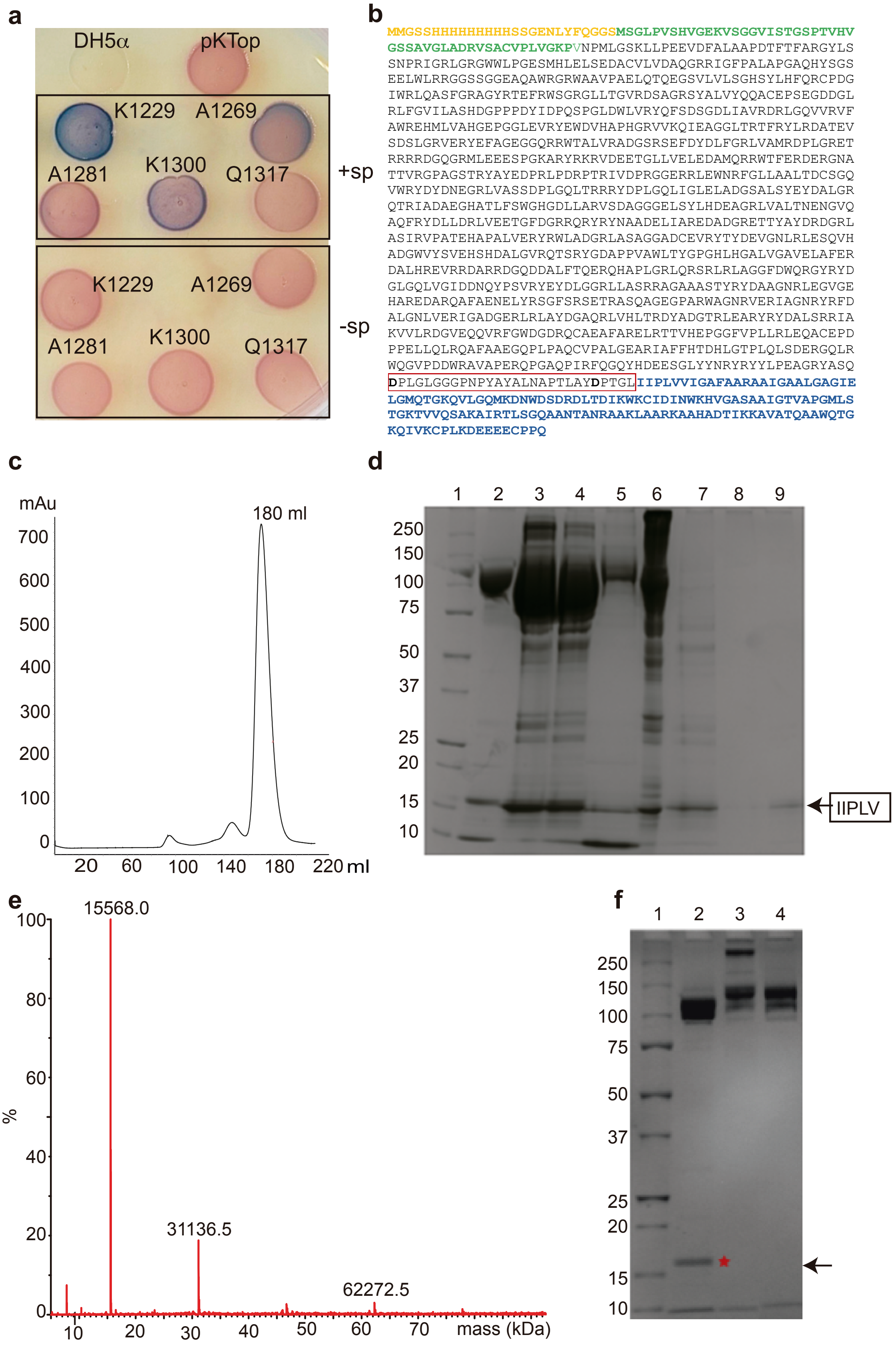

Supplementary Fig. 4 | PhoA-LacZ dual reporter strategy for topology analysis, Tse5/Tse5-CT protein purification analysis, and autoproteolytic activity of Tse5. a. In the absence of a signal

peptide (-sp), the reporter PhoA-LacZα fusion always remains in the cytosol. *E. coli* DH5α cells transformed with spTse5-CT-PhoA-LacZα and Tse5-CT-PhoA-LacZα fusion proteins growing on dual reporter agar plates. In the absence of a signal peptide (-sp), the reporter PhoA-LacZα fusion remains in the cytosol independently of the Tse5-CT fusion point. As a results, the colonies growing on dual reporter LB agar plates are always coloured in red as a result of the β-galactosidase activity of LacZ. **b.** The protein sequence of Tse5 full length construct used in this study for heterologous expression in *E. coli* Lemo21 cells. The construct contains a 9xhis-tag at the N-terminus to facilitate the purification, coloured in yellow. The three predicted domains of Tse5 are indicated in different colours: The N-terminal domain in green, the Rhs domain in black and the Tse5-CT sequence in blue. The conserved motif that forms the active site of a putative aspartyl protease that releases the C-terminal is delimited by a red rectangle, with the two aspartic residues highlighted in bold (D1141 and D1164). **c.** Purified Tse5 behaves in solution as a monodisperse protein. Size exclusion chromatography (Superdex 200 26/600) of the Tse5 full-length protein expressed in *E. coli* Lemo21 cells shows an elution peak at 180 mL, which according to molecular weight standards approximates to a Tse5 monomer (146 kDa). **d.** Tse5-CT can be purified from Tse5 following protein denaturation and precipitation. Sodium dodecyl sulfate-polyacrylamide gel electrophoresis (SDS-PAGE) analysis of Tse5-CT purification (4-20% gel (ExpressPlus™ PAGE Gel, GenScript). Lane 1, molecular weight standard (Precision Plus Protein™ Standards, BIO-RAD); Lane 2, pure Tse5 full length; Lane3, Tse5 dissolved in buffer A with 8M urea; Lane 3, unbound fraction of the nickel resin; Lane 4, 100% B elution of the nickel resin; Lane 5, the pellet of 0.9M ammonium sulphate precipitation; Lane 7, supernatant of 0.9 M ammonium sulphate precipitation; Lane 8, supernatant of wash steps; Lane 9, pure Tse5-CT. The N-terminal sequence of Tse5-CT was confirmed to be **IIPLV** by N-terminal Edman sequencing (sequence indicated in the SDS-PAGE gel). **e.** Mass Spectrometry analysis confirms the identity of purified Tse5-CT. The identity of the purified Tse5-CT protein (see lane 9 in the SDS-PAGE (panel (d)) was confirmed by MS analysis. The major peak corresponds to Tse5-CT (the calculated mass for Tse5-CT is 15571.02 Da), while the other two peaks approximate to the molecular weight of the dimer and trimer, respectively. **f.** Single point mutants of putative catalytic residues D1141A and D1164A are unable to cleave Tse5 to release the Tse5-CT. SDS-PAGE analysis of Tse5, D1141A, and D1164A purifications (4-20% gel (ExpressPlus™ PAGE Gel, GenScript). Line 1, molecular weight standard (Precision Plus Protein™ Standards, BIO-RAD); Lane 2, purified Tse5 full length showing the Tse5-CT above the 15 kDa marker (red star); Lane 3, purified D1141A mutant; Line 4, purified D1164A mutant. Both mutants are unable to cleave the Tse5-CT.

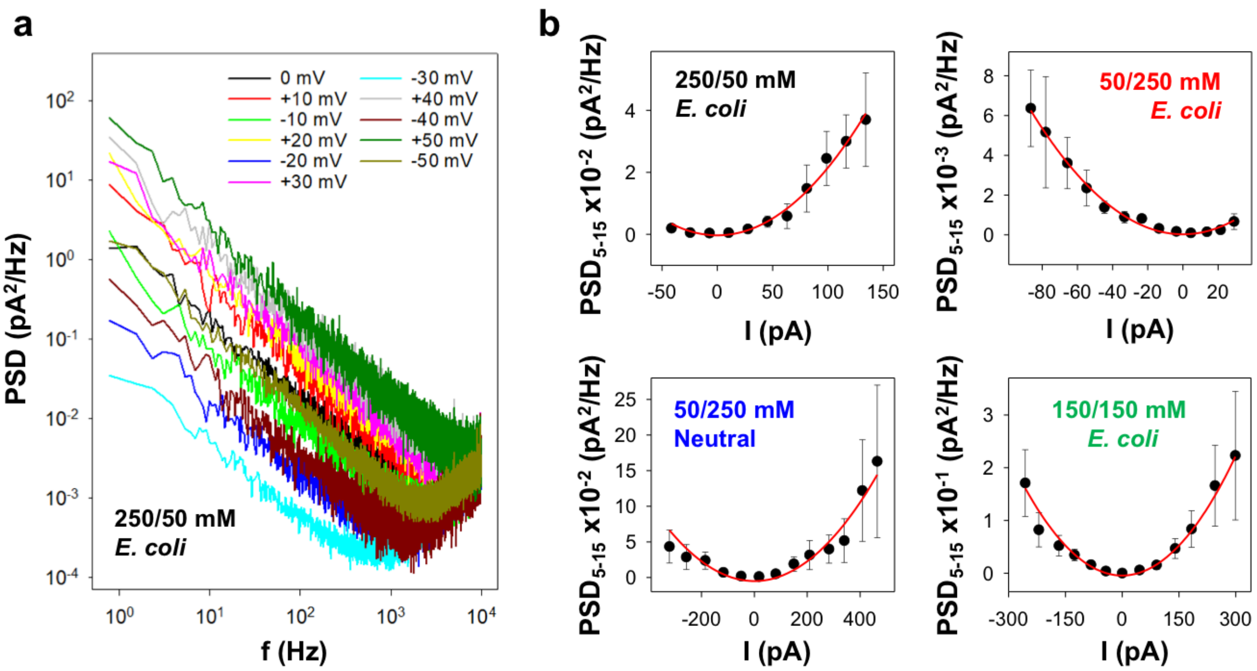

Supplementary Fig. 5 | Tse5-CT-induced currents display 1/f power spectral densities and equilibrium conductance fluctuations. a. Representative PSDs as a function of frequency obtained from Tse5-CT-induced currents in 250/50 mM KCl gradient and using a polar lipid extract from *E. coli* to form the membrane. **b.** Each curve corresponds to a different applied voltage, as indicated**.** Averaged PSD at the 5-15 Hz band as a function of the measured current obtained from PSDs (a) for the different explored conditions, as indicated. Solid lines represent a parabolic fitting.

a b

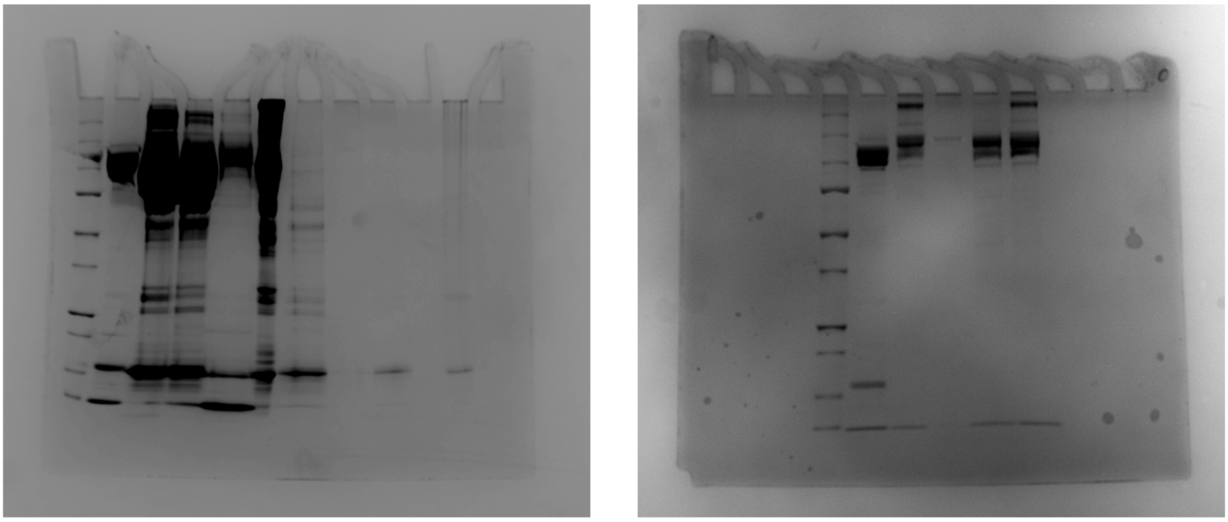

Supplementary Fig. 6 | Uncropped and unedited SDS-gels shown in Supplementary Fig. 3. a. Sodium dodecyl sulfate-polyacrylamide gel electrophoresis (SDS-PAGE) analysis of Tse5-CT purification. **b.** SDS-PAGE analysis of Tse5, D1141A, and D1164A purifications.

Supplementary Note 1 LC-ESI-MS report for Tse5-CT

ETH zürich | University of Zurich | functional genomics center zurich | f·g·c·z

Protein and Proteomics analyses
Functional Genomics Center Zurich
UZH/ETH Zürich
Winterthurerstrasse 190
CH-8057
Dr. Serge Chesnov
+41 44 635 39 50

**Requested analysis:**	**Protein Characterisation**
**Order/Sample:**	24894/ 4 samples
**Customer:**	David Albesa-Jove
**Date:**	26/05/2021
**Analysis ID:**	20210525

Procedure

Due to high sample heterogeneity (proven by direct-infusion ESI-MS), the samples were analysed in an LC-ESI-MS approach.

The samples were 3- resp. 2-fold diluted with 1% TFA and transferred to an autosampler vial for LC/MS. 7 ul resp. 10 ul of sample were injected into an ACQUITY UPLC@ BioResolve-RP-mAb 2.7µ 2.1x150 450 A (Waters, USA) column. For separation and elution on an Acquity UPLC station, a gradient buffer A (0.1% FA in water)/ buffer B (0.1% FA in AN) at a flow rate 200ul/min at 50^0 C over 25 min was applied.

Time	Flow (ml/min)	%A	%B	Curve
Initial	0.2	85	15	Initial
3.0	0.2	45	55	6

20.3	0.2	20	80	6
21.3	0.2	85	15	6
25	0.2	85	15	6

The analysis was performed on a Synapt G2 mass spectrometer directly coupled to the UPLC station.

Mass spectra were acquired in the positive-ion mode by scanning the m/z range from 100 to 4000 da with a scan duration of 1 s and an interscan delay of 0.1s. The spray voltage was set to 3 kV, the cone voltage to 50V, and the source temperature to 80 °C. The data were recorded with the MassLynx 4.2 Software (both Waters, UK). Where possible, the recorded m/z data of single peaks were deconvoluted into mass spectra by applying the maximum entropy algorithm MaxEnt1 (MaxLynx) with a resolution of the output mass 0.5 Da/channel and Uniform Gaussian Damage Model at the half height of 0.5 Da.

Useful links

- Free Scaffold viewer
- FGCZ intranet pages: Frequently Asked Questions
  - FGCZ Quick Scaffold guide (how to access the most relevant Scaffold features)
- Tools for analyses of pull-down experiments
  - String: Protein-Protein interaction networks
- Expasy tools for proteomics
  - PeptideCutter
  - PeptideMass
- Uniprot
- Human repositories
  - NextProt: Exploring the universe of human proteins

  - [ProteomicsDB](): expedite the identification of the human proteome
- [PaxDb](): Protein Abundance Database

Frequently asked questions (FAQs):

Please refer to our FAQ pages within the FGCZ intranet page.

If you do not find an answer to your questions, please contact us using the Comment functionality within your B-Fabric order.

For questions not related to a specific order, please send an email to:

proteomics@fgcz.ethz.ch

FGCZ policies

Unless stated otherwise, all the analyses have been performed under the FGCZ Terms and Conditions.

In case of full or partial publication of the results, please acknowledge the FGCZ for the analyses and the technical support (Functional Genomics Center Zurich (FGCZ), University/ETH Zurich).

All the unused samples are discarded 4 week after analyses.

Data

The following pages show the results of the requested analyses.

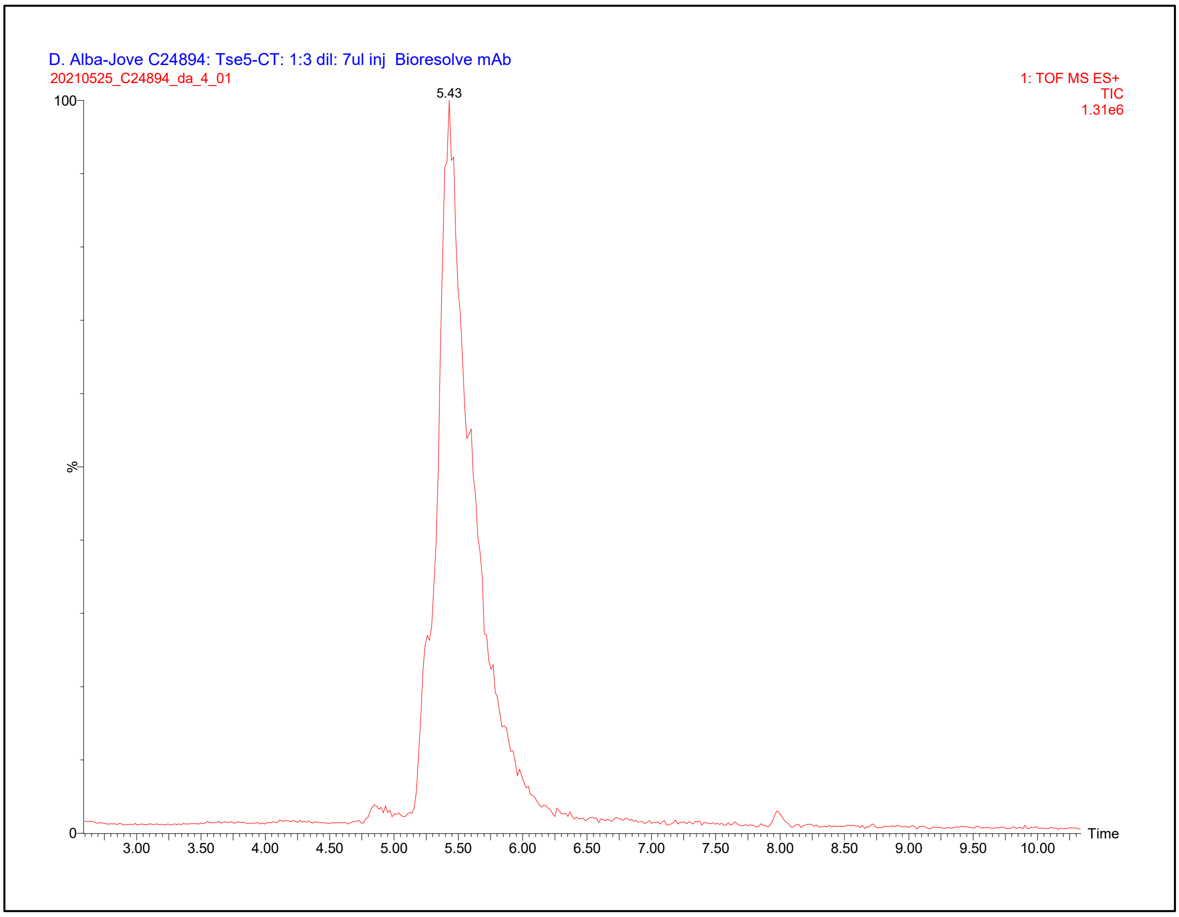

D. Alba-Jove C24894: Tse5-CT: 1:3 dil: 7ul inj Bioresolve mAb
20210525_C24894_da_4_01
1: TOF MS ES+
TIC
1.31e6
5.43
100
%
0
3.00 3.50 4.00 4.50 5.00 5.50 6.00 6.50 7.00 7.50 8.00 8.50 9.00 9.50 10.00
Time

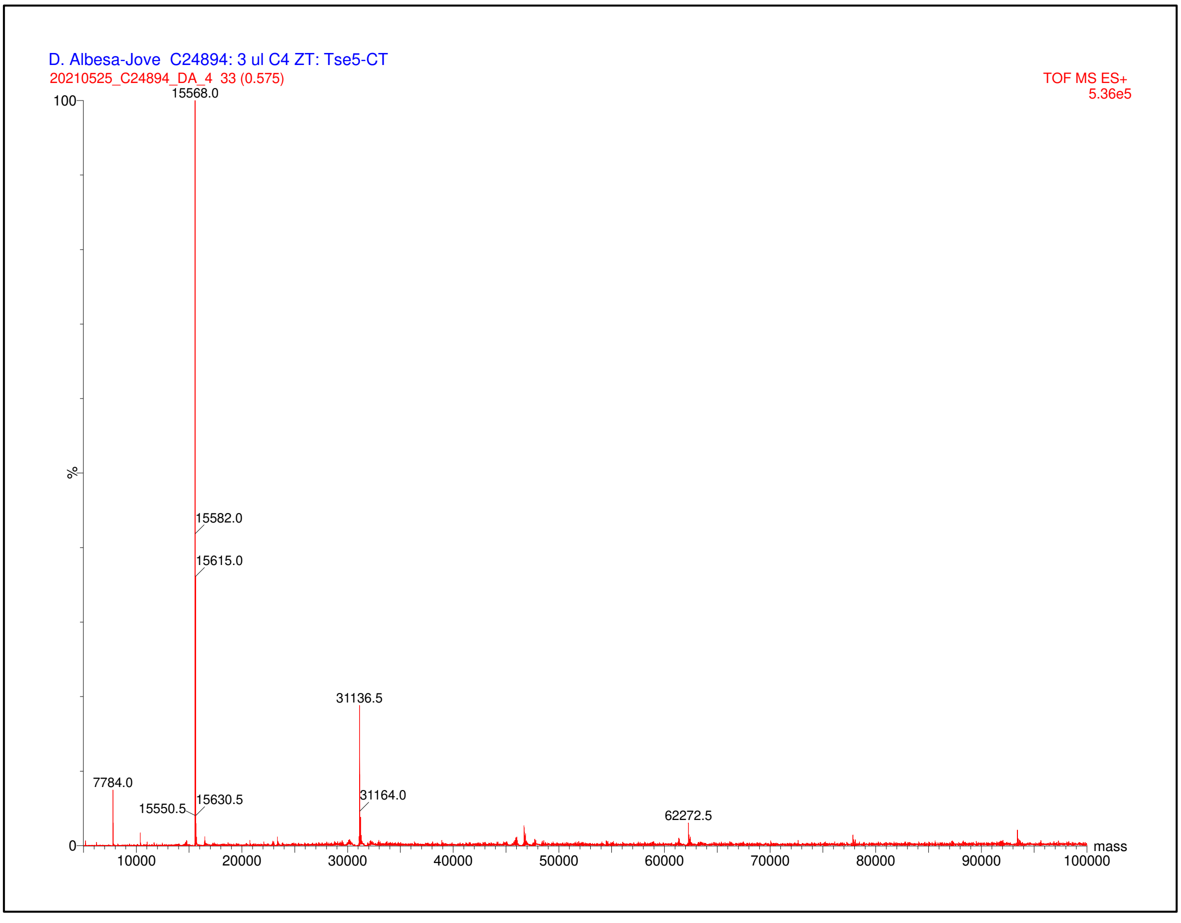

D. Albesa-Jove C24894: 3 ul C4 ZT: Tse5-CT
20210525_C24894_DA_4 33 (0.575)
TOF MS ES+
5.36e5
15568.0
15582.0
15615.0
31136.5
7784.0
15550.5
15630.5
31164.0
62272.5
100
%
0
10000 20000 30000 40000 50000 60000 70000 80000 90000 100000
mass

Supplementary Note 2 N-terminal sequencing report

SequencePro™

QUIMICA DE PROTEINAS (CIB-CSIC)
Ramiro de Maeztu, 9
28040 Madrid

PNA-SQP004-F02

SAMPLE INFORMATION

Sample Name:	David Albesa_Cterminal	Std Amount:	8.000 pmols
ID Code:		Sample Amount:	0.000 pmols
		Detector Scale:	0.005 AUFS
Comments:	IIPLV		

SEQUENCER INFORMATION

Name:	PROCISE	Model Number:	494
Method:	Pulsed liquid PVDF mod	Cartridge:	D
Operator:	Javier Varela		

Wednesday, March 09, 2022 09:49:15

David Albesa_Cterminal - 08Mar2022 10-09-34 - Page 1 of 8

Cycle 1:Blank 1

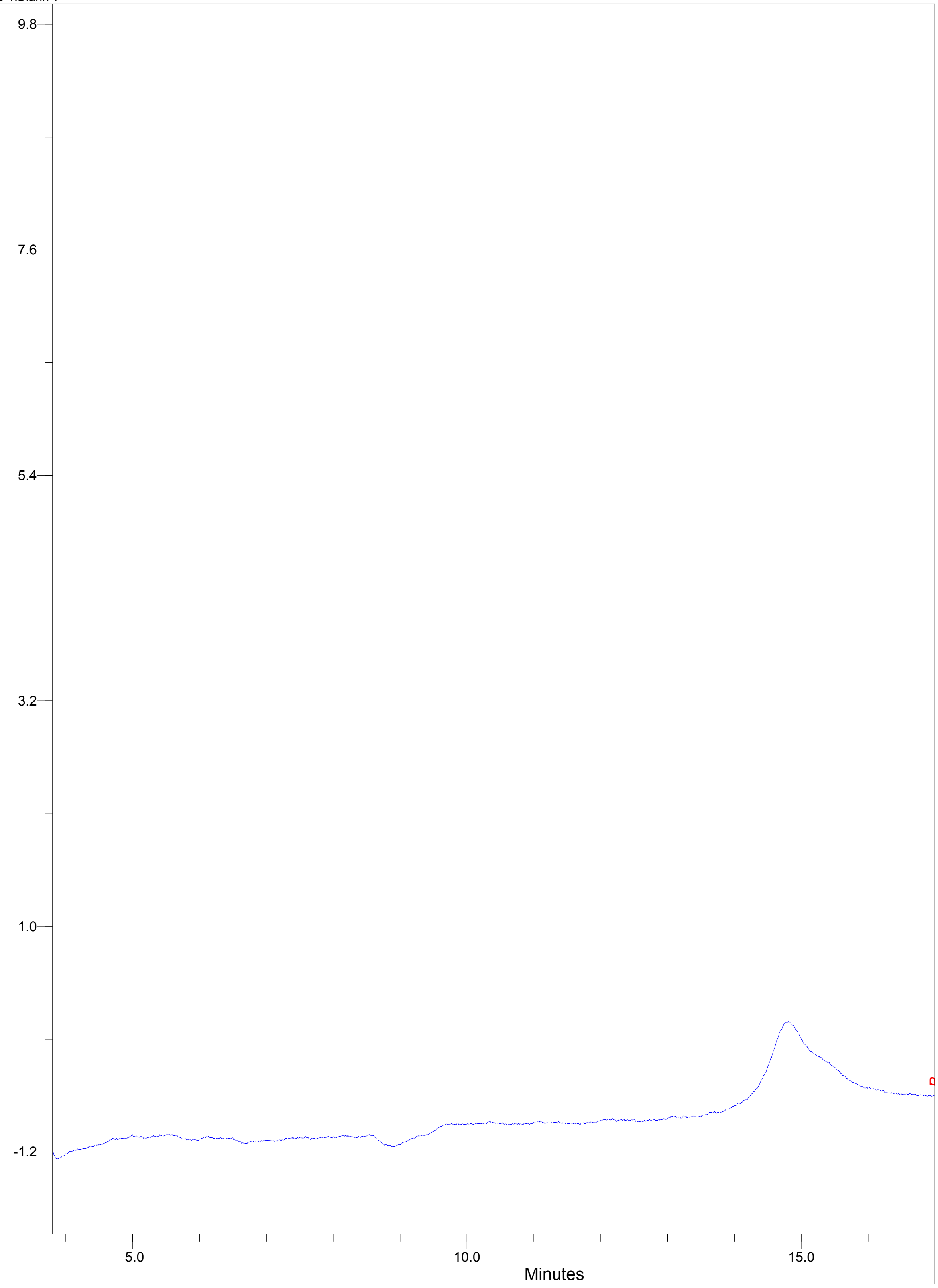

Cycle 2:

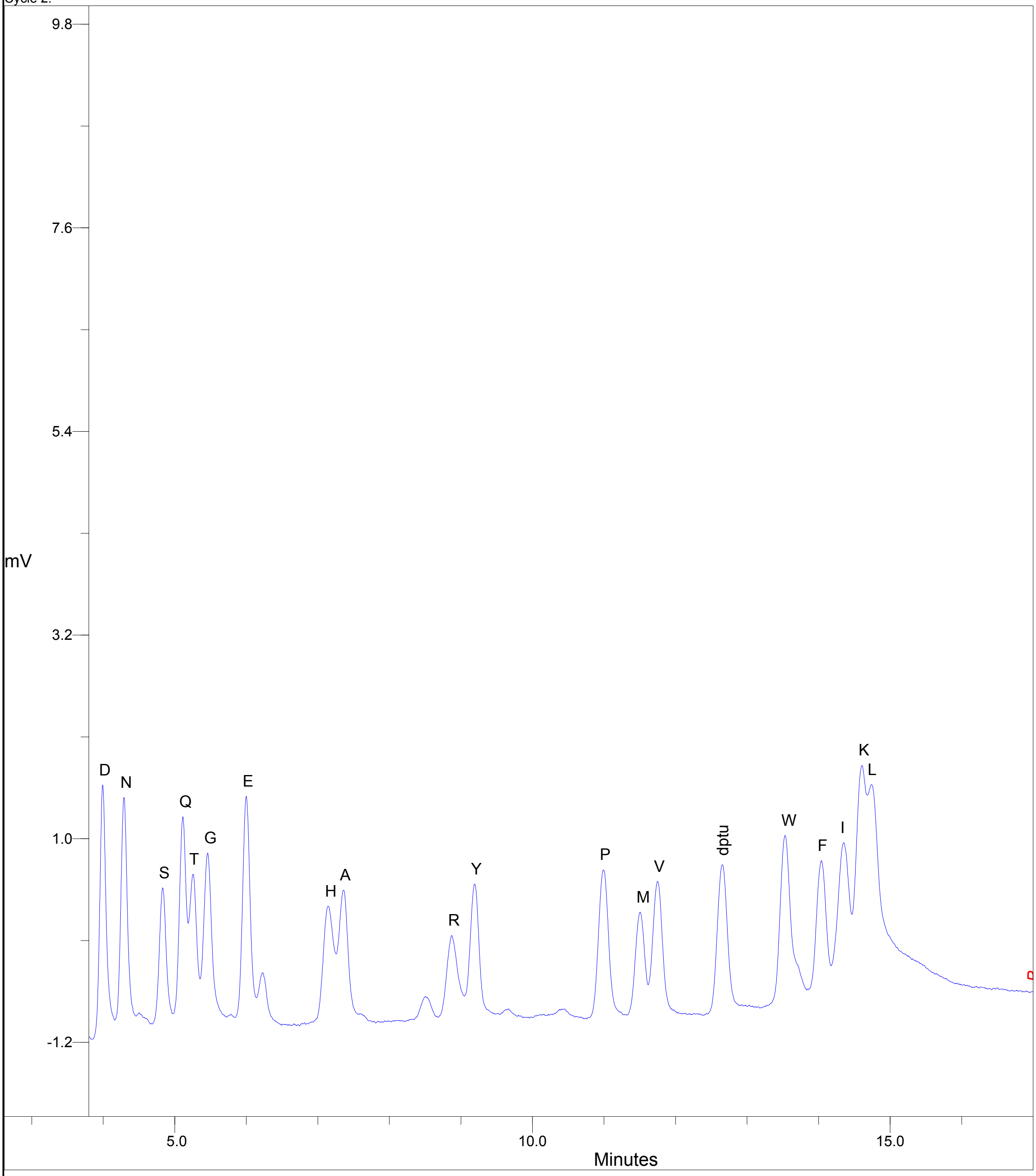

PEAK ID	R.TIME (mins)	C.TIME (mins)	HEIGHT (mV)	PMOL HT	PEAK ID	R.TIME (mins)	C.TIME (mins)	HEIGHT (mV)	PMOL HT
D	3.99	3.99	2.741	8.000	Y	9.19	9.19	1.453	8.000
N	4.29	4.29	2.591	8.000	P	10.99	10.99	1.605	8.000
S	4.83	4.83	1.582	8.000	M	11.50	11.50	1.129	8.000
Q	5.11	5.11	2.333	8.000	V	11.75	11.75	1.453	8.000
T	5.25	5.25	1.704	8.000	dptu	12.66	12.66	1.575	8.000
G	5.46	5.46	1.922	8.000	W	13.53	13.53	1.814	8.000
E	6.00	6.00	2.505	8.000	F	14.04	14.04	1.469	8.000
H	7.15	7.15	1.232	8.000	I	14.35	14.35	1.615	8.000
A	7.36	7.36	1.374	8.000	K	14.61	14.61	2.415	8.000
R	8.87	8.87	0.908	8.000	L	14.74	14.74	2.186	8.000

Cycle 3:

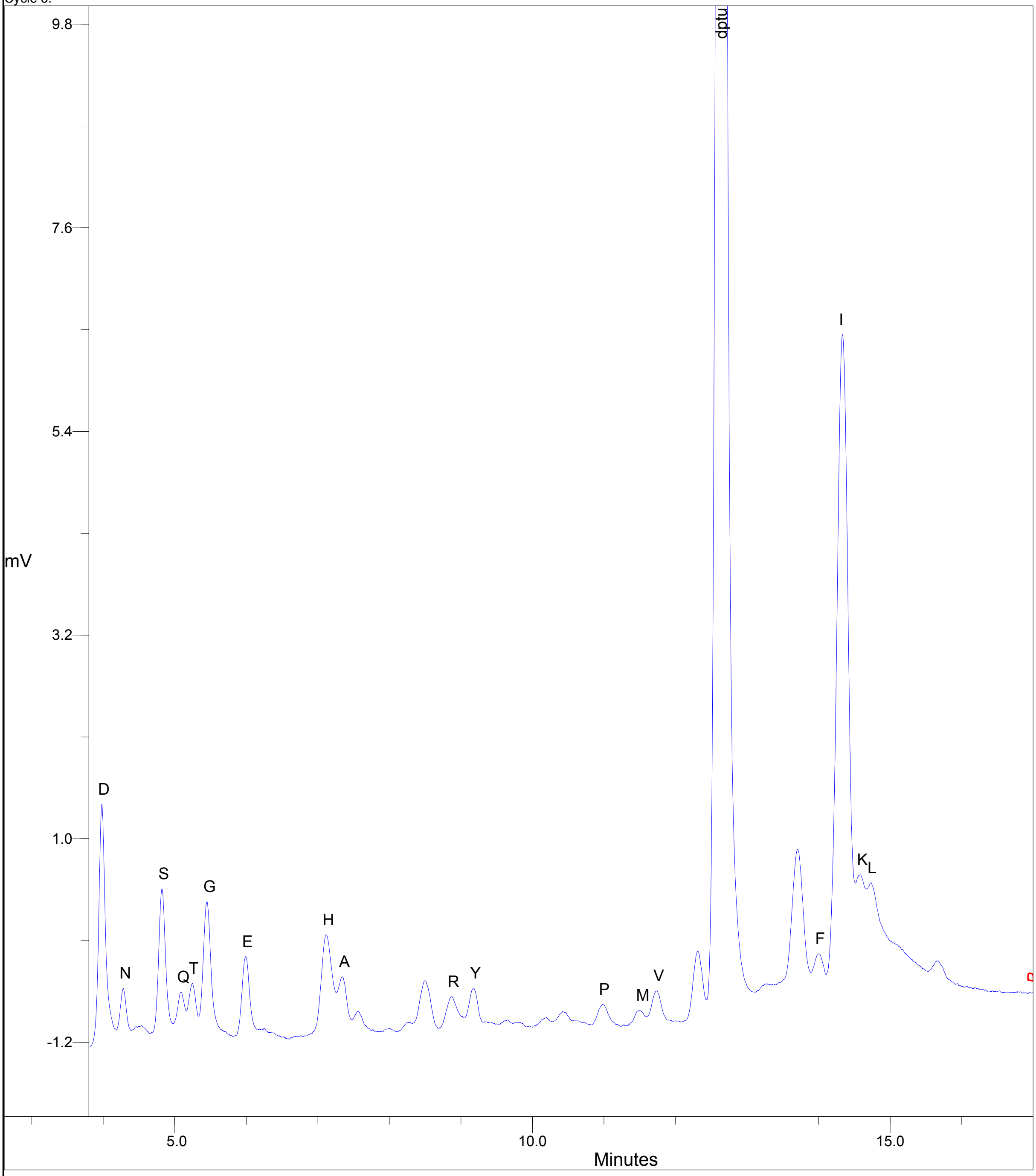

PEAK ID	R.TIME (mins)	C.TIME (mins)	HEIGHT (mV)	PMOL HT
D	3.98	3.99	2.616	7.636
N	4.28	4.29	0.612	1.888
S	4.82	4.83	1.657	8.382
Q	5.08	5.11	0.529	1.815
T	5.25	5.25	0.613	2.878
G	5.45	5.46	1.486	6.186
E	5.99	6.00	0.858	2.740
H	7.12	7.15	1.089	7.067
A	7.35	7.36	0.618	3.600
R	8.87	8.87	0.329	2.903

PEAK ID	R.TIME (mins)	C.TIME (mins)	HEIGHT (mV)	PMOL HT
Y	9.18	9.19	0.399	2.196
P	10.98	10.99	0.241	1.203
M	11.50	11.50	0.157	1.115
V	11.74	11.75	0.348	1.915
dptu	12.64	12.66	27.562	139.970
F	14.00	14.04	0.580	3.157
I	14.33	14.35	7.242	35.866
K	14.59	14.61	0.921	3.051
L	14.73	14.74	1.192	4.361

Cycle 4:

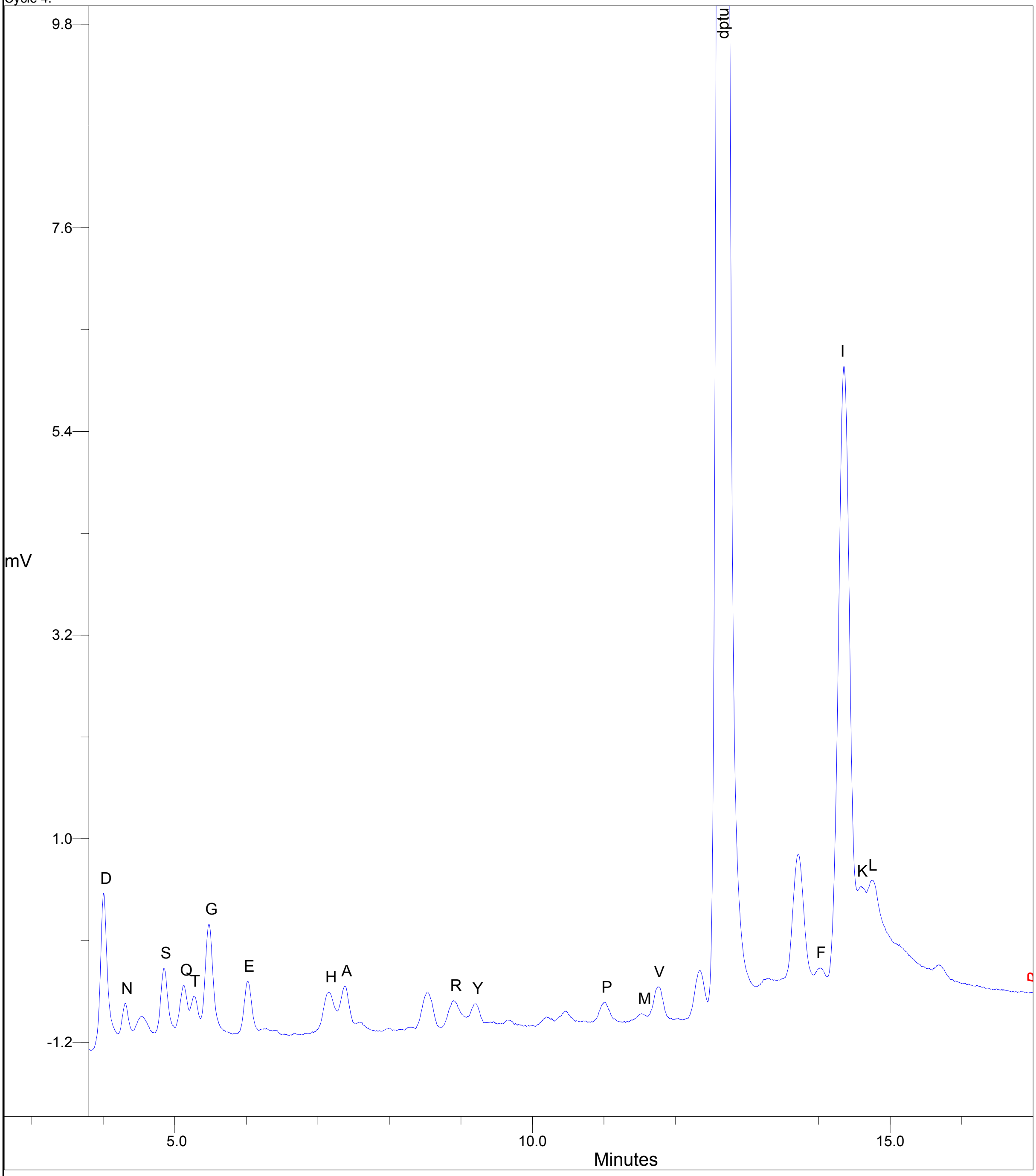

PEAK ID	R.TIME (mins)	C.TIME (mins)	HEIGHT (mV)	PMOL HT
D	4.01	3.99	1.688	4.928
N	4.30	4.29	0.480	1.481
S	4.85	4.83	0.824	4.170
Q	5.13	5.11	0.624	2.141
T	5.27	5.25	0.492	2.309
G	5.47	5.46	1.261	5.249
E	6.02	6.00	0.606	1.934
H	7.15	7.15	0.449	2.914
A	7.38	7.36	0.506	2.948
R	8.90	8.87	0.281	2.477

PEAK ID	R.TIME (mins)	C.TIME (mins)	HEIGHT (mV)	PMOL HT
Y	9.21	9.19	0.221	1.218
P	11.02	10.99	0.216	1.078
M	11.53	11.50	0.090	0.638
V	11.75	11.75	0.373	2.052
dptu	12.66	12.66	35.895	182.288
F	14.02	14.04	0.383	2.085
I	14.35	14.35	6.850	33.921
K	14.59	14.61	0.664	2.201
L	14.75	14.74	1.165	4.263

Cycle 5:

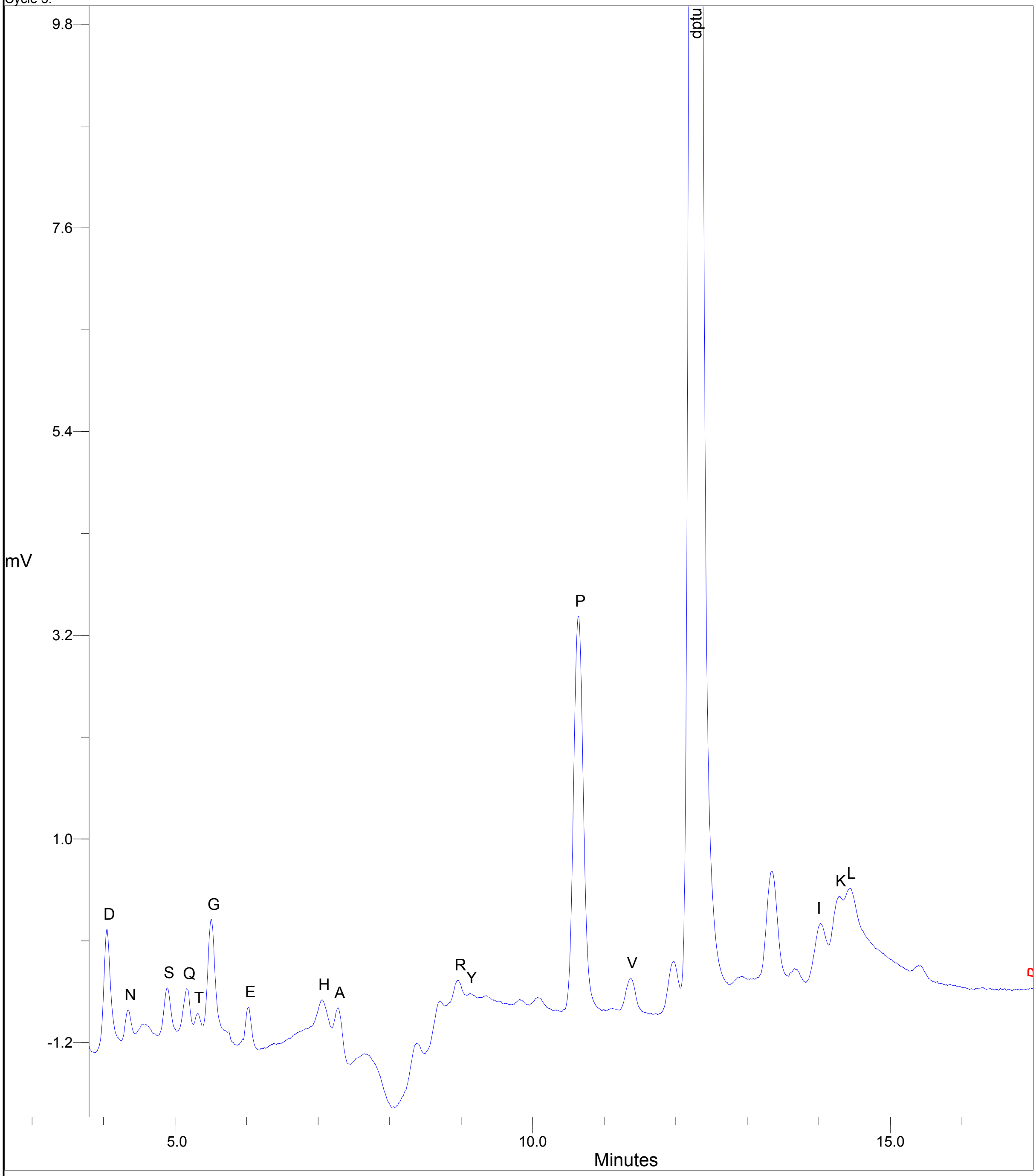

PEAK ID	R.TIME (mins)	C.TIME (mins)	HEIGHT (mV)	PMOL HT
D	4.05	3.99	1.361	3.973
N	4.35	4.29	0.532	1.643
S	4.89	4.83	0.846	4.279
Q	5.16	5.11	0.876	3.006
T	5.32	5.25	0.635	2.979
G	5.50	5.46	1.675	6.974
E	6.03	6.00	0.799	2.553
H	7.05	7.15	1.024	6.650
A	7.28	7.36	0.969	5.642
R	8.96	8.87	0.537	4.727

PEAK ID	R.TIME (mins)	C.TIME (mins)	HEIGHT (mV)	PMOL HT
Y	9.12	9.19	0.304	1.671
P	10.64	10.99	4.281	21.339
V	11.37	11.75	0.386	2.125
dptu	12.28	12.66	38.326	194.635
I	14.02	14.35	0.784	3.882
K	14.28	14.61	1.055	3.494
L	14.44	14.74	1.125	4.117

Cycle 6:

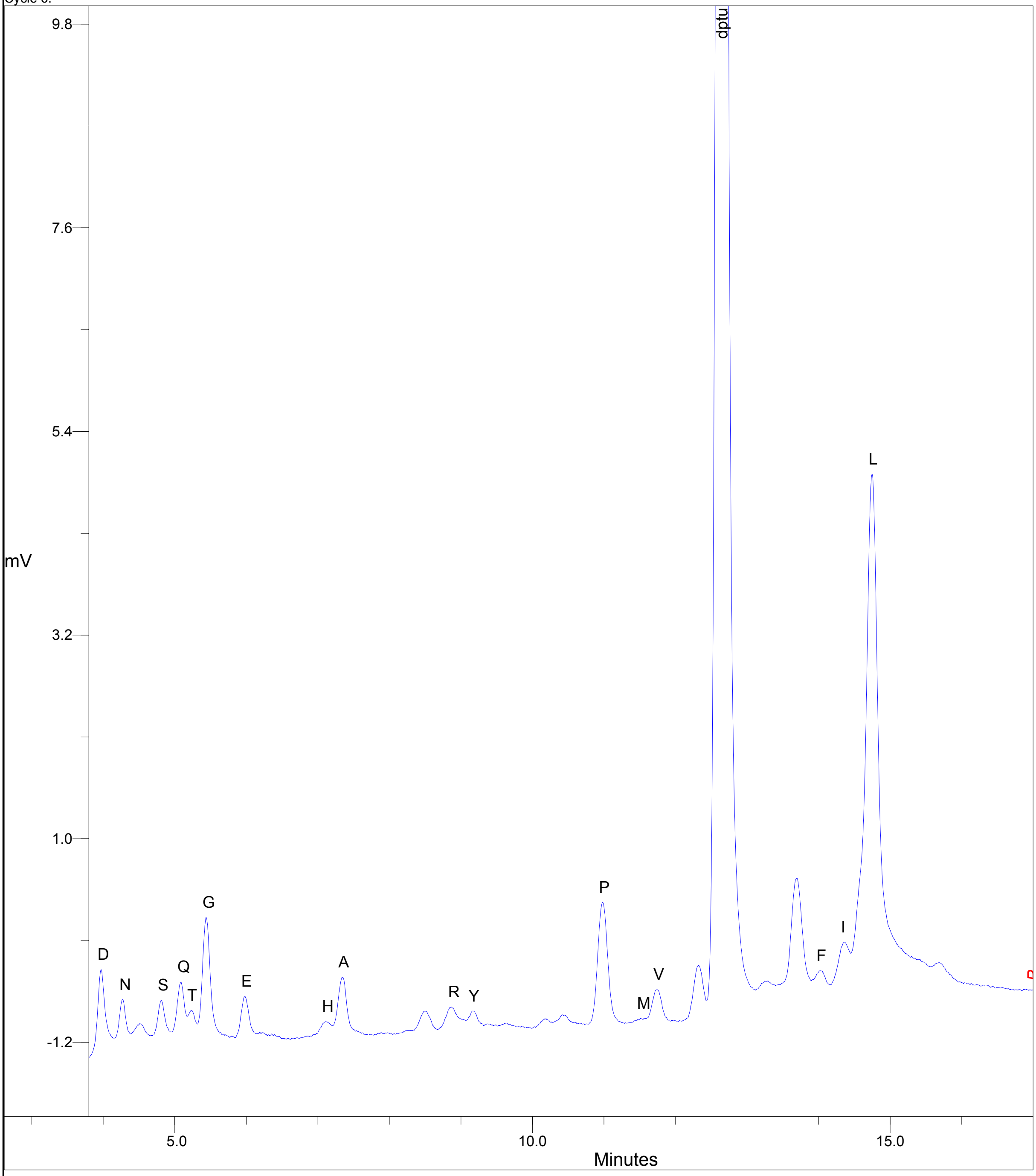

PEAK ID	R.TIME (mins)	C.TIME (mins)	HEIGHT (mV)	PMOL HT
D	3.97	3.99	0.930	2.714
N	4.28	4.29	0.569	1.757
S	4.81	4.83	0.491	2.485
Q	5.09	5.11	0.654	2.243
T	5.22	5.25	0.330	1.550
G	5.44	5.46	1.309	5.449
E	5.98	6.00	0.429	1.370
H	7.11	7.15	0.175	1.133
A	7.34	7.36	0.646	3.760
R	8.87	8.87	0.261	2.299
Y	9.16	9.19	0.205	1.126
P	10.98	10.99	1.316	6.560
M	11.51	11.50	0.050	0.355
V	11.74	11.75	0.354	1.950
dptu	12.65	12.66	34.146	173.410
F	14.02	14.04	0.349	1.898
I	14.36	14.35	0.622	3.079
L	14.75	14.74	5.640	20.635

Cycle 7:

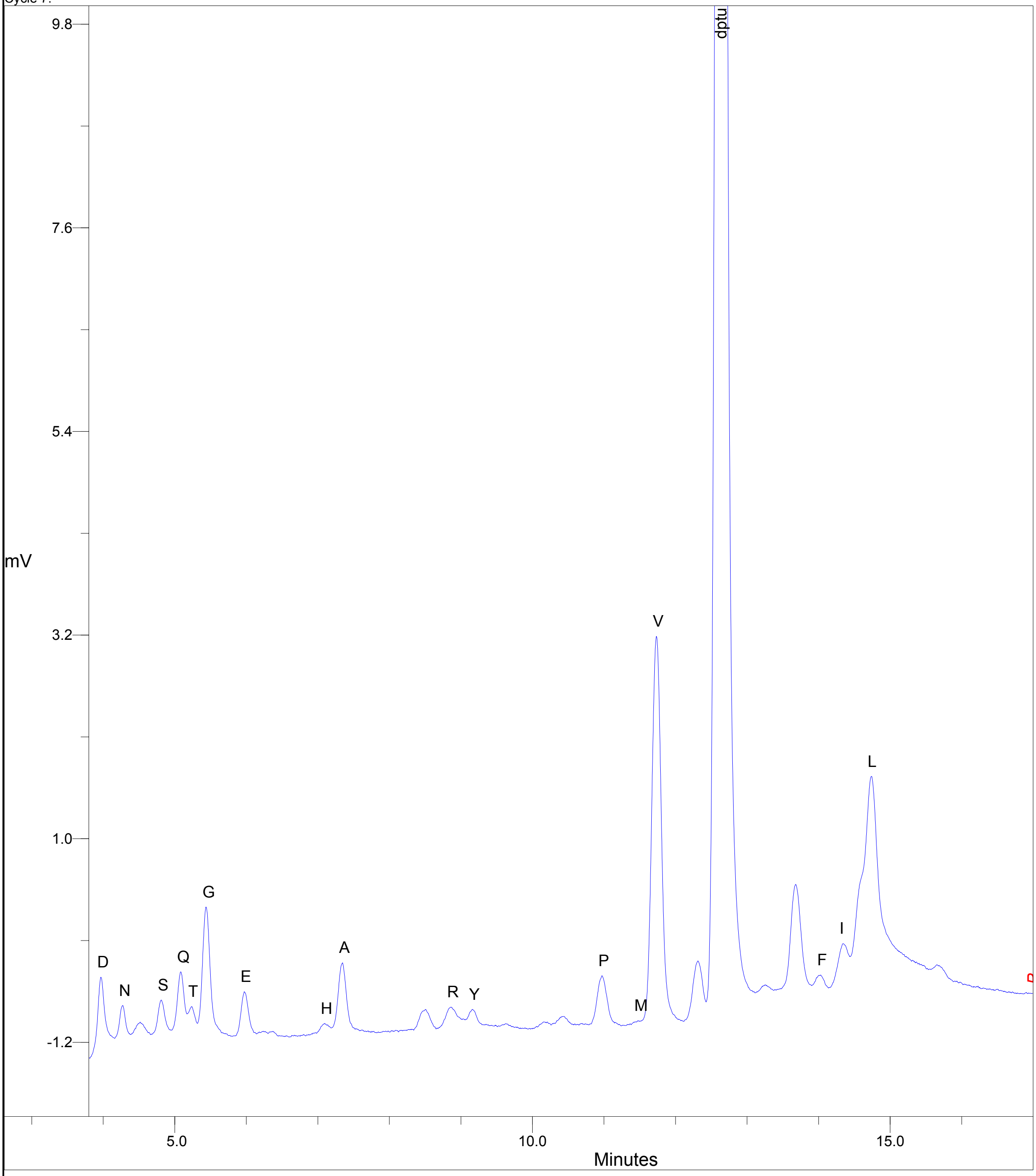

PEAK ID	R.TIME (mins)	C.TIME (mins)	HEIGHT (mV)	PMOL HT
D	3.97	3.99	0.831	2.427
N	4.27	4.29	0.435	1.342
S	4.81	4.83	0.378	1.910
Q	5.08	5.11	0.686	2.352
T	5.24	5.25	0.313	1.471
G	5.44	5.46	1.392	5.797
E	5.97	6.00	0.485	1.549
H	7.09	7.15	0.116	0.756
A	7.35	7.36	0.754	4.390
R	8.86	8.87	0.231	2.032

PEAK ID	R.TIME (mins)	C.TIME (mins)	HEIGHT (mV)	PMOL HT
Y	9.16	9.19	0.191	1.050
P	10.97	10.99	0.540	2.690
M	11.48	11.50	0.045	0.318
V	11.73	11.75	4.183	23.036
dptu	12.63	12.66	33.180	168.502
F	14.03	14.04	0.161	0.878
I	14.34	14.35	0.436	2.158
L	14.74	14.74	2.157	7.892

Supplementary references

1. Bernal, P. *et al.* A novel stabilization mechanism for the type VI secretion system sheath. *Proc. Natl. Acad. Sci. U. S. A.* **118**, e2008500118 (2021).
2. Lambertsen, L., Sternberg, C. & Molin, S. Mini-Tn7 transposons for site-specific tagging of bacteria with fluorescent proteins. *Environ. Microbiol.* **6**, 726–732 (2004).
3. Schlegel, S. *et al.* Optimizing membrane protein overexpression in the Escherichia coli strain Lemo21(DE3). *J. Mol. Biol.* **423**, 648–659 (2012).
4. Martínez-García, E., Nikel, P. I., Aparicio, T. & de Lorenzo, V. Pseudomonas 2.0: Genetic upgrading of P. putida KT2440 as an enhanced host for heterologous gene expression. *Microb. Cell Fact.* **13**, 159 (2014).
5. Whitney, J. C. *et al.* Genetically distinct pathways guide effector export through the type VI secretion system. *Mol. Microbiol.* **92**, 529–542 (2014).
6. Karimova, G., Robichon, C. & Ladant, D. Characterization of YmgF, a 72-residue inner membrane protein that associates with the Escherichia coli cell division machinery. *J. Bacteriol.* **91**, 333–346 (2009).
7. Calles, B., Goñi-Moreno, Á. & Lorenzo, V. Digitalizing heterologous gene expression in Gram-negative bacteria with a portable ON/OFF module. *Mol. Syst. Biol.* **15**, e8777 (2019).
8. Silva-Rocha, R. *et al.* The Standard European Vector Architecture (SEVA): A coherent platform for the analysis and deployment of complex prokaryotic phenotypes. *Nucleic Acids Res.* **41**, D666–D675 (2013).
