# Supplementary material for: The P. aeruginosa effector Tse5 forms membrane pores disrupting the membrane potential of intoxicated bacteria
Source: Commun Biol. 2022 Nov 5;5:1189. doi: 10.1038/s42003-022-04140-y (PMC9637101; doi:10.1038/s42003-022-04140-y)
Supplement: Supplementary file 2 — Description of Additional Supplementary Files [file 42003_2022_4140_MOESM2_ESM.pdf]

Description of Additional Supplementary Files

File name: Supplementary Data 1
Description: Supplementary Data 1 contains numeric data for charts included within the article.
